# Molecular dynamics reveals insight into how N226P and H227Y mutations affect maltose binding in the active site of α-glucosidase II from European honeybee, *Apis mellifera*

**DOI:** 10.1371/journal.pone.0229734

**Published:** 2020-03-03

**Authors:** Panachai Punnatin, Chanpen Chanchao, Surasak Chunsrivirot

**Affiliations:** 1 Faculty of Science, Department of Biology, Chulalongkorn University, Pathumwan, Bangkok, Thailand; 2 Faculty of Science, Structural and Computational Biology Research Unit, Department of Biochemistry, Chulalongkorn University, Pathumwan, Bangkok, Thailand; 3 Faculty of Science, Department of Biochemistry, Chulalongkorn University, Pathumwan, Bangkok, Thailand; University of Akron, UNITED STATES

## Abstract

European honeybee, *Apis mellifera*, produces α-glucosidase (HBGase) that catalyzes the cleavage of an α-glycosidic bond of the non-reducing end of polysaccharides and has potential applications for malt hydrolysis in brewing industry. Characterized by their substrate specificities, HBGases have three isoforms including HBGase II, which prefers maltose to sucrose as a substrate. Previous study found that the catalytic efficiency of maltose hydrolysis of N226P mutant of HBGase II was higher than that of the wild type (WT), and the catalytic efficiency of maltose hydrolysis of WT was higher than those of H227Y and N226P-H227Y mutants. We hypothesized that N226P mutation probably caused maltose to bind with better affinity and position/orientation for hydrolysis than WT, while H227Y and N226P-H227Y mutations caused maltose to bind with worse affinity and position/orientation for hydrolysis than WT. Using this hypothesis, we performed molecular dynamics on the catalytically competent binding conformations of maltose/WT, maltose/N226P, maltose/H227Y, and maltose/N226P-H227Y complexes to elucidate effects of N226P and H227Y mutations on maltose binding in HBGase II active site. Our results reasonably support this hypothesis because the N226P mutant had better binding affinity, higher number of important binding residues, strong and medium hydrogen bonds as well as shorter distance between atoms necessary for hydrolysis than WT, while the H227Y and N226P-H227Y mutants had worse binding affinities, lower number of important binding residues and strong hydrogen bonds as well as longer distances between atoms necessary for hydrolysis than WT. Moreover, results of binding free energy and hydrogen bond interaction of residue 227 support the role of H227 as a maltose preference residue, as proposed by previous studies. Our study provides important and novel insight into how N226P and H227Y mutations affect maltose binding in HBGase II active site. This knowledge could potentially be used to engineer HBGase II to improve its efficiency.

## Introduction

Honeybee α-glucosidase (HBGase), produced by *Apis mellifera*, is an exo-type carbohydrase that catalyzes the cleavage of an α-glycosidic bond of the non-reducing end of polysaccharides. HBGase can be classified based on their substrate specificities and locations in internal organs into three isoforms HBGase I, II and III [1]. Previous study found that HBGase II preferred maltose as a substrate, while HBGase III preferred sucrose as a substrate [2]. They also proposed residue 227 as a substrate preference residue. Specifically, H227 is a maltose preference residue in HBGase II, while Y227 is a sucrose preference residue in HBGase III. Recently, the results of molecular dynamics study of HBGase III also supported the role of Y227 as a sucrose preference residue [3]. Moreover, they found that when Y227 was mutated to H227, the Y227H mutant preferred binding maltose to sucrose, supporting the role of H227 as a maltose preference residue.

**Fig 1:**
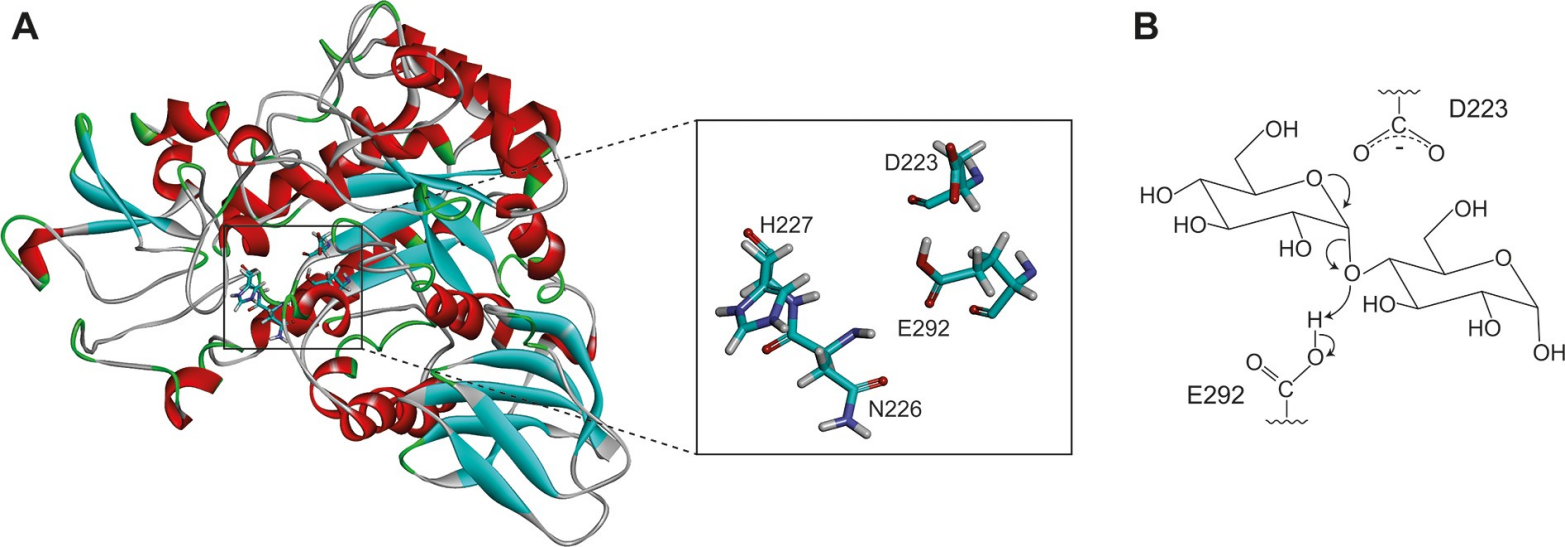
(A) Homology model of *Apis mellifera* HBGase II with D223, E292, N226 and H227 shown in licorice. (B) The first step of the proposed hydrolysis reaction of α-glycosidic bond via an oxocarbenium ion intermediate.

HBGase II belongs to glycoside hydrolase family 13 and it can be found in ventriculus and hemolymph [1]. The previous study proposed D223 and E292 (Fig 1A) to be its catalytic residues [4]. Potential applications of HBGase II include production of isomalto-oligosaccharides (IMOs) [5]. Due to their functions in mammalian metabolic processes and its low calories, IMOs are widely used as prebiotics, food additives and feed ingredients [6,7]. Moreover, HBGase II could potentially be used in brewing industry, where *α*‐glucosidase is used for malt hydrolysis [8,9].

Many carbohydrate-degrading enzymes, including glucosidases, lysozymes, and amylases, were proposed to catalyze a reaction via the oxocarbenium ion intermediate mechanism, where the carboxyl and carboxylate group cooperate in the hydrolytic reaction. Fig 1B shows the first step of the proposed mechanism. The oxygen atom of the glycosidic bond abstracts the proton from E292, promoting the formation of oxocarbenium ion intermediate and releasing a hydrolytic product. The carboxylate group of D223 was proposed to promote the formation and stabilization of oxocarbenium ion intermediate [10]. This mechanism is supported by the α-secondary kinetic isotope effects reported in hydrolysis by lysozyme, glucoamylases, and α-glucosidases [10]. In addition, this mechanism is suitable for the reaction of both “retained” and “inverted” enzyme [10].

Previous study by Ngiwsara et al. found that the N226P mutation of HBGase II from *Apis mellifera* increased the catalytic efficiency of maltose hydrolysis as compared to that of the wild type (WT) at 310 K and pH 5.5, while the H227Y mutation and N226P-H227Y mutation decreased the catalytic efficiency of maltose hydrolysis [2]. The catalytic efficiency (*k*_cat_/*K*_m_) of the N226P mutant was approximately 418% of that of WT, indicating that the N226P mutant could efficiently hydrolyze maltose better than WT. This result suggests its potential use for malt hydrolysis in brewing industry. On the contrary, the catalytic efficiencies of H227Y mutant (H227Y) and N226P-H227Y mutant (N226P-H227Y) were approximately 59% and 42% of that of WT, respectively. However, molecular level understanding on how these mutations affect the binding of maltose in the active site of this enzyme and subsequently the catalytic efficiency was limited.

In this study, homology models of HBGase II and its N226P, H227Y and N226P-H227Y mutants were created. The catalytically competent binding conformations of maltose in the active site of WT and three mutants were identified. Then, molecular dynamics simulations of maltose/WT, maltose/N226P, maltose/H227Y, and maltose/N226P-H227Y complexes were performed at the experimental temperature and pH to elucidate the effects of these mutations on the binding of maltose in the active site of these enzymes.

## Materials and methods

### Structure preparation

Sequence of *Apis mellifera* α-glucosidase II (GenBank ID: BAE86927.1) was retrieved from the National Center for Biotechnology Information (NCBI). To construct the homology model of α-glucosidase II from *Apis mellifera*, SWISS-MODEL server [11–13] was employed using the crystal structure of *Erwinia rhapontici* isomaltulose synthase (PDB ID: 4HPH [14]) as a template, which was the template used in our previous study in *Apis mellifera* HBGase III [3]. To evaluate the quality of the homology model, RAMPAGE server [15] was used to generate a Ramachandran plot. This homology model has reasonable quality, since most residues of this homology model are in favored region (92.8%) and allowed region (5.2%) as shown in S1 Fig. The proposed catalytic residues such as D223 and E292 were also found in suitable positions for hydrolysis. The structure was protonated at the experimental pH of 5.5, using the H++ server [16–18]. ACE and NME groups were employed to cap the N-terminus and C-terminus, respectively. AMBER ff14SB force field was used to assign the atom types and force field parameters, and the LEaP module in AMBER18 [19] was used to add hydrogen and missing atoms. E292 was also protonated, following the proposed mechanism from previous study (Fig 1B). The LEaP module was used to mutate N226 to P226 and H227 to Y227 to create N226P and H227Y mutants, respectively. The N226P-H227Y mutant was also created using the LEaP module.

### Identification of the catalytically competent binding conformations and molecular dynamics (MD) simulations

The catalytically competent binding conformations of the maltose/WT, maltose/N226P, maltose/H227Y, and maltose/N226P-H227Y complexes were identified using the method modified from Sitthiyotha et al. [20]. The structure of maltose was obtained from *Halomonas* sp. H11 α-glucosidase (PDB ID: 3WY4 [21]). Their atom types and force field parameters were assigned by the GLYCAM06j-1 force field [22]. Autodock vina [23] with the grid box of 20×20×20 Å^3^ and 1 Å spacing centered on the center of mass of D223, E292 and D354 was used to predict the binding conformations of maltose in the active sites of WT and mutants. Moreover, the crystal maltose was redocked into the active site of *Halomonas* sp. H11 α-glucosidase (3WY4 [21]) to validate if Autodock vina and its parameters were appropriate for our systems. We found that the best docked and crystal binding conformation were reasonably similar with RMSD value of 0.33 Å. As a result, Autodock vina and its corresponding parameters were used to predict maltose binding conformations. For each system, 20 independent docking runs were performed, resulting in nine possible binding conformations per each run and the total of 180 binding conformations. To identify the catalytically competent binding conformations of each complex, the docked conformations were screened by the distance necessary for hydrolysis that was defined as the distance between an oxygen atom of the glycosidic bond of maltose and an acidic hydrogen atom of the acid-base catalytic residue, E292 (O4-HE distance). The binding conformations that had the distance within 5 Å were retained and were then clustered by their structural similarities as measured by the RMSD values of their heavy atoms using the MMTSB Tool Set [24]. A centroid of each cluster was selected from the binding conformation that was most similar to the average structure of all members of each cluster. For a cluster with more than 10 members, the second and third most similar conformations to the average structure were also selected (S1–S4 Tables). All selected binding conformations were used to construct the complex in an isometric truncated octahedral box of TIP3P water molecules with the buffer distance of 13 Å by the LEaP module. To neutralize all systems, chloride ions (Cl^-^) were added. Structural minimizations and MD simulations were performed by AMBER 18. Five-step procedure was used to minimize all systems as described below. For each minimization step, 1,000 steps of steepest descent and 1,000 steps of conjugate gradient were conducted. Initially, non-hydrogen atoms of proteins were restrained with the force constant of 10 kcal/(mol Å^2^). Then, the backbones of the proteins were restrained with the force constants of 10, 5 and 1 kcal/(mol Å^2^), respectively. Ultimately, the whole system was minimized without applying any restraints. All systems were then simulated under periodic boundary condition using GPU (CUDA) version of PMEMD module of AMBER18 [25–27]. To constrain all bonds with hydrogen atoms, the SHAKE algorithm [28] was employed; therefore, a simulation time step of 0.002 ps could be used. The cutoff distance of 12 Å was employed for non-bonded interactions. To compute the long-range electrostatic interaction, the particle mesh Ewald method was employed. The Langevin dynamics technique with a collision frequency of 1.0 ps^-1^ was used to regulate the temperature. All systems were heated from 0 K to 310 K (the experimental temperature) during 200 ps in the NVT ensemble, while restraining the backbone with a force constant of 10 kcal/(mol Å^2^). Then, with no restraint, these systems were equilibrated for 300 ps at 310 K in the NVT ensemble. Finally, the systems were further simulated for 60 ns in the NPT ensemble at 310 K and 1 atm.

In this study, the catalytically competent binding conformation was defined as the binding conformation that had an appropriate orientation and distance for hydrolysis to occur. This binding conformation should have the position of oxygen of the glycosidic bond of maltose that was not too far from that of the acidic hydrogen of E292. Therefore, the O4-HE distances of all binding conformations were measured during the simulations. For the maltose/WT, maltose/N226P, and maltose/H227Y complexes, the binding conformations with the O4-HE distance greater than 5 Å were eliminated, and the binding conformation with stable and reasonable values of RMSD and lowest O4-HE distance during the simulations was selected to be the catalytically competent binding conformation of each system. For the maltose/N226P-H227Y complex, since all representative binding conformations have the O4-HE distance greater than 5 Å, the representative binding conformation with the O4-HE distance more than 5 Å but less than or equal to 7 Å were considered, instead. With the assumption that catalytically competent binding conformations should be the ones, where maltose stably bind in the active site of the enzyme, the binding conformation with the lowest fluctuation of O4-HE distance and the lowest all atom and maltose RMSD value was selected as the catalytically competent binding conformation of the maltose/N226P-H227Y complex.

To determine whether maltose binding affects HBGase II and maltose structures, MD simulations of free maltose and free enzymes were also performed. For free enzymes, the same protocol as that of the complexes was used. In the case of maltose, the crystal structure of maltose was submerged in an isometric truncated octahedral box of TIP3P water molecules with the buffer distance of 20 Å. Two-step procedure was used to minimize the system as described below. For each minimization step, 1,000 steps of steepest descent and 1,000 steps of conjugate gradient were conducted. Initially, non-hydrogen atoms of maltose were restrained with the force constant of 10 kcal/(mol Å^2^). Then, the whole system was minimized without applying any restraints. Finally, MD simulations were performed using the same protocol as that of complexes.

For analyses, RMSD values were used to measure the stabilities of all systems. As the RMSD values of all systems were stable around 40–60 ns, these trajectories were used for further analyses. To measure the binding affinity between maltose and HBGase II, molecular mechanics/generalized born surface area (MM/GBSA) method [29,30] was performed to calculate total binding free energies and per-residue binding free energy decomposition.

The energy contributions of each residue of the four complexes was represented as a four-dimensional vector. Residues of HBGase II with contribution (≠ 0 kcal/mol) to maltose binding in at least one system were selected for hierarchical clustering analysis using R 3.6.0 [31]. Manhattan distance was used to calculate the differences between each vector:

$$Distance(a,b)=\sum_{i}\left|a_{i}-b_{i}\right|$$,

where i denotes each dimension of a and b. For clustering, the Ward’s minimum variance method was used, and the hierarchical clustering tree graph was visualized by iTOL [32].

Hydrogen bonds between maltose and the enzymes were measured employing CPPTRAJ [33] under these criteria: (i) a proton donor-acceptor distance ≤ 3.00 Å and (ii) a donor-H-acceptor bond angle ≥ 135°. Strong and medium hydrogen bonds were defined as the hydrogen bonds with occupation > 75% and 50–75%, respectively.

## Results and discussion

### System stabilities

**Fig 2:**
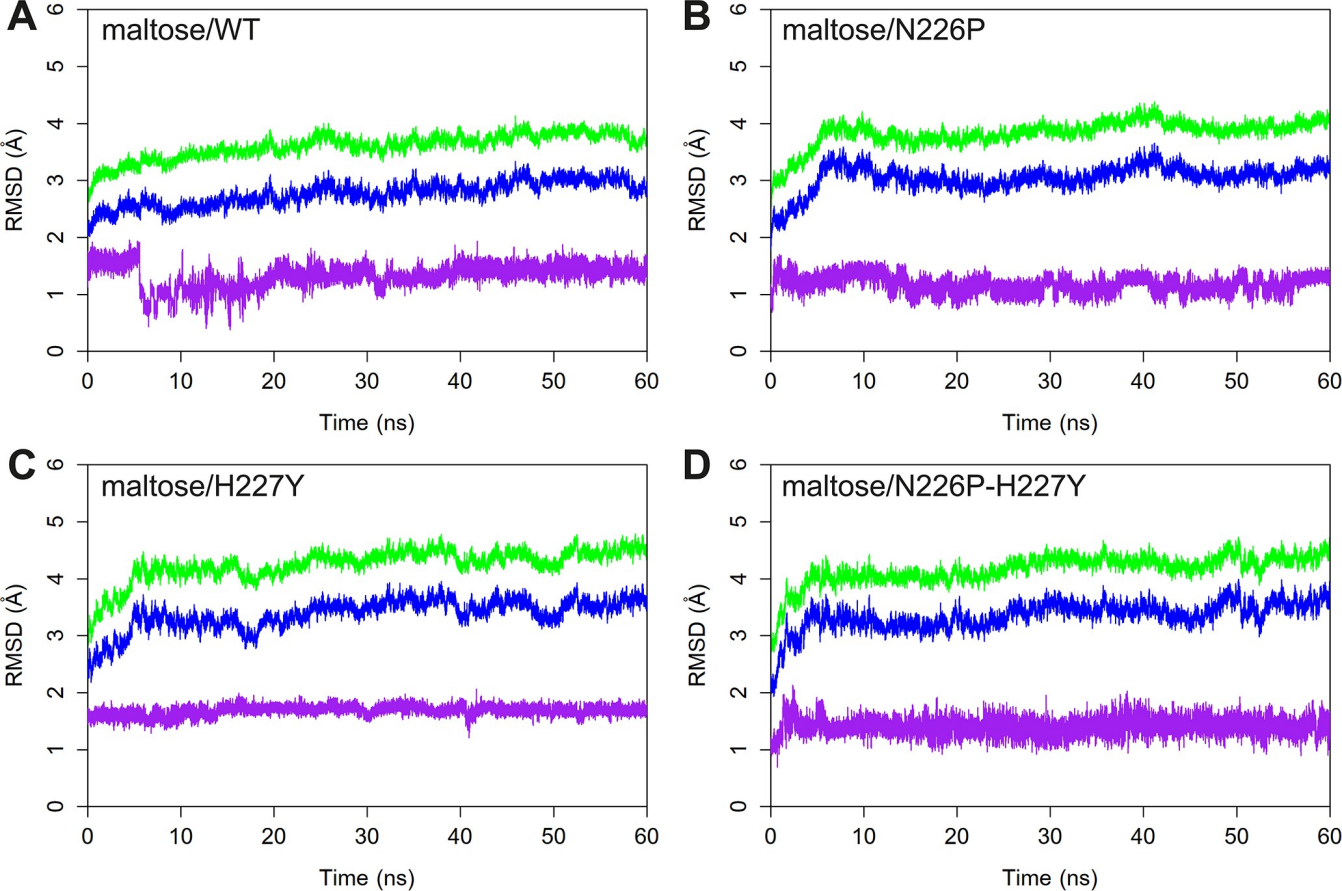
RMSD plots of A) maltose/WT B) maltose/N226P C) maltose/H227Y and D) maltose/N226P-H227Y complexes. The RMSD values of all atoms, backbone atoms, and maltose are shown in green, blue and purple, respectively.

To determine the stabilities of the catalytically competent binding conformations of the maltose/WT, maltose/N226P, maltose/H227Y, and maltose/N226P-H227Y complexes, the RMSD values of all atoms, the backbone atoms of the enzymes, and maltose atoms were measured, using the minimized structures as the references. As shown in Fig 2, the simulations were likely to reach equilibrium around 40 ns. Therefore, the 40–60 ns trajectories were used for further analyses.

### Distances between atoms necessary for hydrolysis

**Fig 3:**
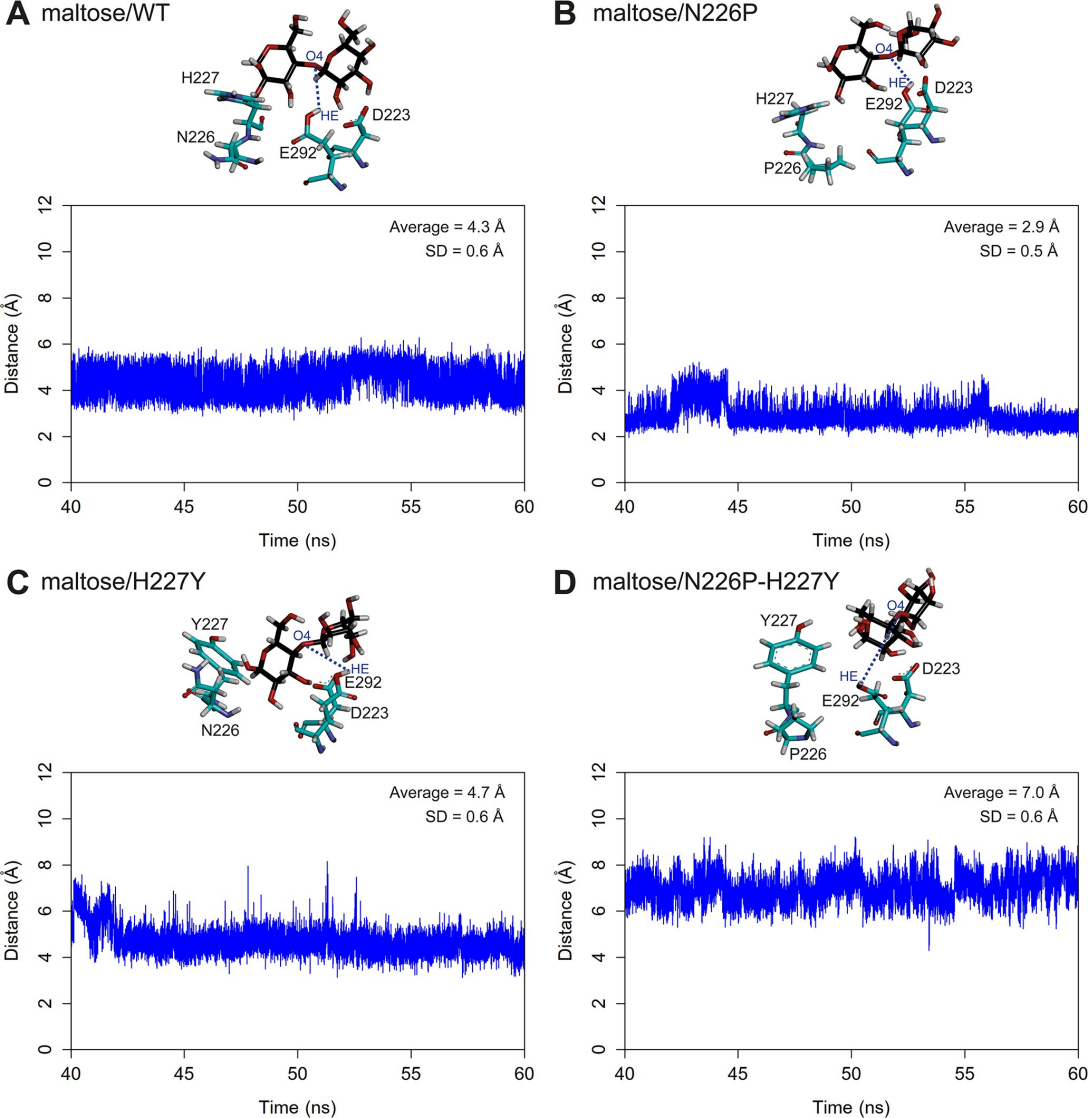
Distances between the oxygen atom of the glycosidic bond of maltose and the acidic hydrogen of E292 of A) maltose/WT B) maltose/N226P C) maltose/H227Y and D) maltose/N226P-H227Y complexes. Residues 223, 292, 226 and 227 as well as maltose are shown in stick representation and colored by atom types, where carbon atoms of amino acid atoms are green and those of maltose are black.

The distances between the oxygen atom of the glycosidic bond of maltose and the acidic hydrogen of the acid-base catalytic residue (E292) of all systems were measured as shown in Fig 3. The O4-HE distances of all systems are stable during 40–60 ns simulations. Moreover, the ranking of the average O4-HE distances is N226P (2.9) < WT (4.3) < H227Y (4.7) < N226P-H227Y (7.0). When the distance between atoms necessary for hydrolysis is reasonable and not too far, an enzyme is more likely to catalyze the reaction effectively because the glycosidic bond of maltose is able to abstract the acidic hydrogen of the acid-base catalytic residue, following the proposed mechanism in the previous study (Fig 1B). The average O4-HE distance of N226P is shorter than that of WT, suggesting that the N226P mutant could potentially catalyze the reaction better than WT. However, the average O4-HE distances of N227Y and N226P-H227Y mutants are longer than that of WT, suggesting that these two mutants could catalyze the reaction worse than WT. The ranking of the O4-HE distances also support the ranking of the experimental catalytic efficiency of maltose hydrolysis (N226P > WT > H227Y > N226P-H227Y) from previous study.

### Conformations of free enzymes and maltose

To determine the effects of maltose binding on HBGase II and maltose structure, MD of free maltose and free enzymes were also performed. The backbone atom RMSD values of HBGase II of the maltose complexes of WT, N226P, H227Y, and N226P-H227Y with respect to the free enzyme are 0.50, 1.12, 1.24, and 0.43 Å, respectively, indicating that the conformations of the free enzyme and substrate-enzyme complexes are quite similar (S2–S5 Figs).

In terms of maltose, as shown in S6 Fig, the conformations of maltose in complexes are not significantly different from that of the free maltose. The heavy atom RMSD values of maltose in WT, N226P, H227Y, and N226P-H227Y complexes with respect to the free maltose are 0.50, 1.12, 1.24, and 0.43 Å, respectively.

### Binding free energy calculations

To determine if the calculated binding affinities is correlated with the experimental ranking of the catalytic efficiency of maltose hydrolysis from the previous study, binding free energies of each complex during the 40–60 ns trajectories were calculated using the MM/GBSA method as shown in Table 1. The binding free energies of the maltose/WT, maltose/N226P, maltose/H227Y, and maltose/N226P-H227Y complexes are -15.3±2.6, -28.4±0.7, -12.6±1.1, and -3.9±3.2 kcal/mol, respectively.

**Table 1:** Binding free energies (kcal/mol) and their components of the maltose/WT, maltose/N226P, maltose/H227Y, and maltose/N226P-H227Y complexes.

System	ΔE_vdW_	ΔE_ele_	ΔH ^(a)^	ΔG_pol_	ΔG_np_	ΔG_solv_ ^(b)^	ΔS_tot_	-TΔS_tot_	ΔG_bind_ ^(c)^	SEM of ΔG_bind_
**Maltose/WT**	-36.5	-82.8	-119.3	85.4	-6.4	79.0	-0.080	24.9	-15.4	2.6
**Maltose/N226P**	-38.6	-113.5	-152.1	102.1	-6.4	95.7	-0.090	28.0	-28.4	0.7
**Maltose/H227Y**	-31.6	-82.1	-113.7	81.7	-5.1	76.5	-0.079	24.6	-12.6	1.1
**Maltose/N226P-H227Y**	-27.9	-76.7	-104.6	80.6	-5.2	75.5	-0.081	25.2	-3.9	3.2

a) ΔH = ΔE_vdW_+ ΔE_ele_

b) ΔG_solv_ = ΔG_pol_+ ΔG_np_

c) ΔG_bind_ = ΔE_vdW_+ΔE_ele_+ΔG_solv_-TΔS_tot_

In terms of the components of binding free energies, the main component contributing to maltose binding affinity of all systems is the electrostatic interaction term (ΔE_ele_) as it has the most favorable values ranging from -113.5 to -76.7 kcal/mol. The van der Waals energy terms (ΔE_vdW_) and non-polar solvation terms (ΔG_np_) also contribute to favorable interactions for maltose binding. However, the polar solvation terms (ΔG_pol_) contribute to unfavorable interaction for maltose binding because they have positive values ranging from 80.6 to 102.1 kcal/mol.

### Per-residue binding free energy decompositions

**Fig 4:**
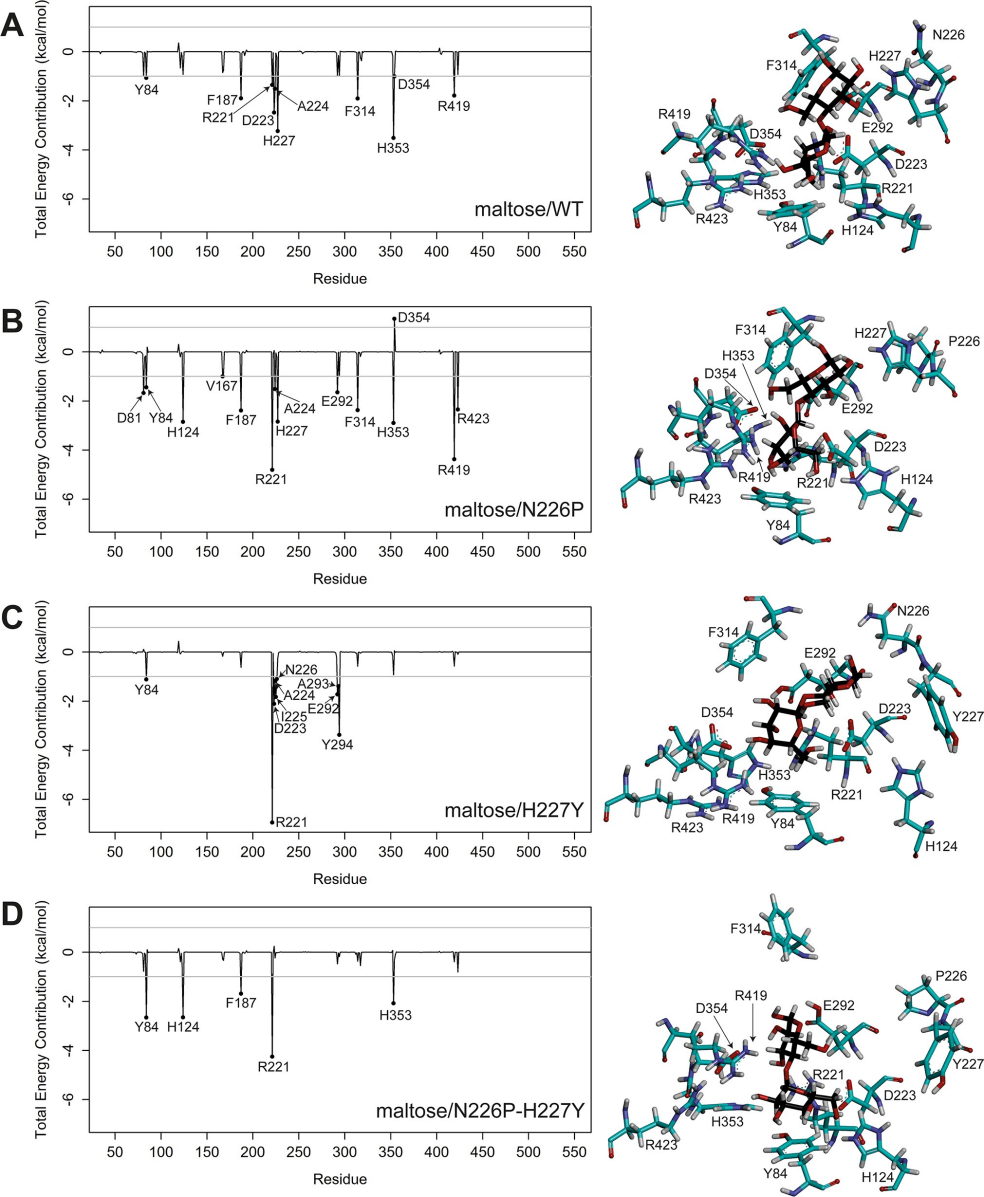
Per-residue decomposition of binding free energy contributions of A) maltose/WT, B) maltose/N226P, C) maltose/H227Y, and D) maltose/N226P-H227Y complexes. Residues 223, 292, 226, 227 and some important binding residues as well as maltose are shown in stick representation and colored by atom types, where carbon atoms of amino acid atoms are green and those of maltose are black.

To identify important binding residues that provide major contributions to the calculated binding free energies, the values of free energy decomposition on a per residue basis ($\Delta G_{\mathrm{bind}}^{\mathrm{residue}}$) were calculated as shown in Fig 4, and per-residue free energy decompositions of binding residues that were defined as residues within 5 Å of maltose of the minimized structure were given in S5–S8 Tables. In this study, an important binding residue, shown in Table 2, was defined to be a residue that contributes to the binding free energy better than -1 kcal/mol. Overall, the ranking of the numbers of important binding residues for maltose-HBGase II complexes is N226P (13) > WT (10) > H227Y (9) > N226P-H227Y (5), and it is in reasonable agreement with the experimental ranking of the catalytic efficiencies of maltose hydrolysis. The important binding residues for all systems are Y84 and R221, indicating their importance in maltose binding of the active sites of WT and the mutants.

**Table 2:** Important binding residues for all maltose-HBGase II systems.

System	Important binding residues
**Maltose/WT**	Y84, F187, R221, D223, A224, H227, F314, H353, D354, R419
**Maltose/N226P**	D81, Y84, H124, V167, F187, R221, A224, H227, E292, F314, H353, R419, R423
**Maltose/H227Y**	Y84, R221, D223, A224, I225, N226, E292, A293, Y294
**Maltose/N226P-H227Y**	Y84, H124, F187, R221, H353

### Hierarchical clustering of per-residue energy contributions

**Fig 5:**
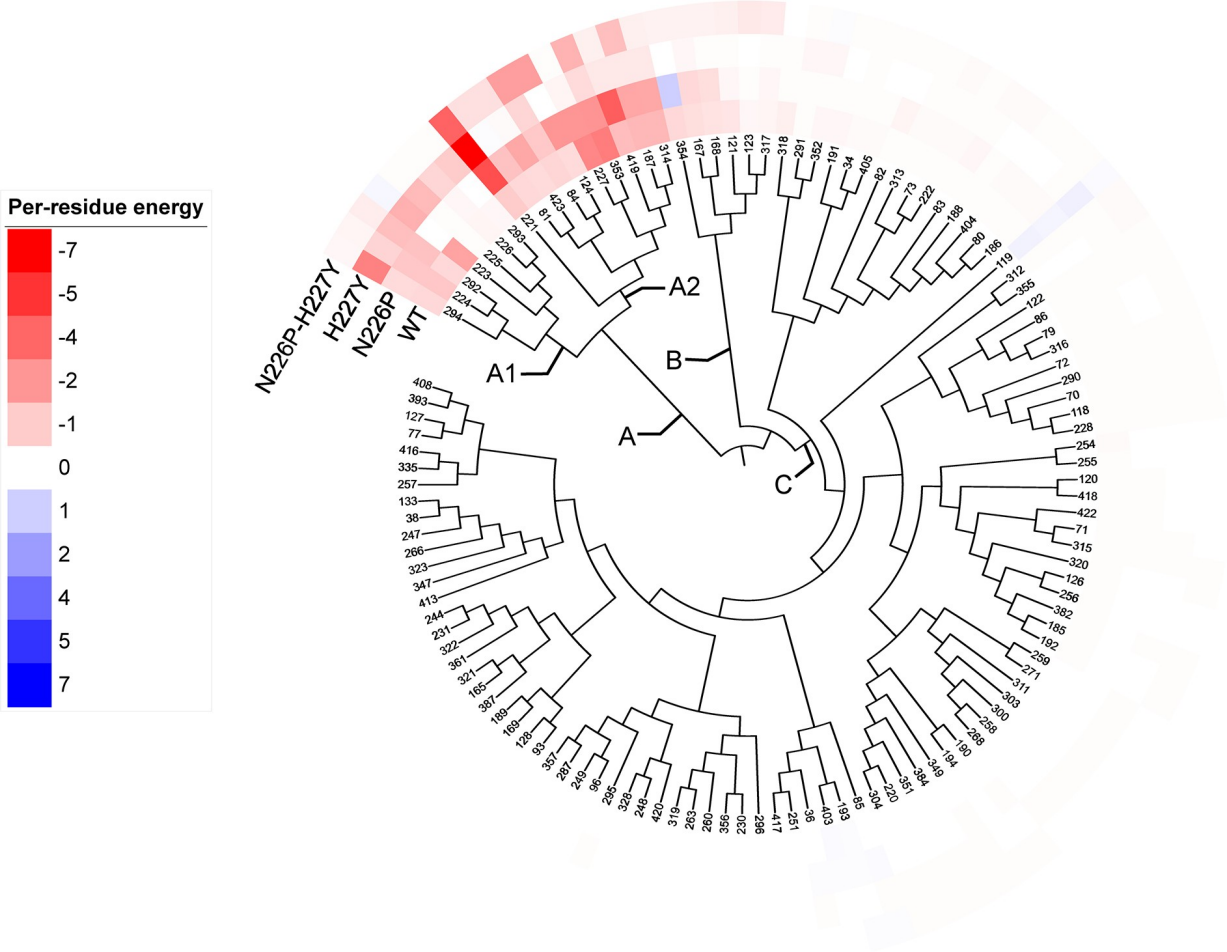
Hierarchical clustering tree of 122 residues of HBGase II with contributions to maltose binding in at least one system by their per-residue energy contributions. Per-residue energy contributions of -7.0 and 7.0 kcal/mol were set as exact red and exact blue, respectively. The colors fade to white towards 0 kcal/mol.

122 residues of HBGase II with contribution (≠ 0 kcal/mol) to maltose binding in at least one system were selected for hierarchical clustering analysis. As shown in Fig 5, three distinct residue groups (A, B, and C) were identified. The energy contributions of residues in group A (D81, Y84, H124, F187, R221, D223, A224, I225, N226P, H227Y, E292, A293, Y294, F314, H353, R419, R423) are consistently higher across all systems than those in group B and C. Moreover, energy contributions of residues in subgroup A2 (D81, Y84, H124, F187, R221, H227Y, F314, H353, R419, R423) are higher than those in subgroup A1 (D223, A224, I225, N226P, E292, A293, Y294). Therefore, residues in subgroup A1 and A2 could be considered as hot spot residues with relatively strong and strong contributions, respectively.

### Hydrogen bond interactions

**Fig 6:**
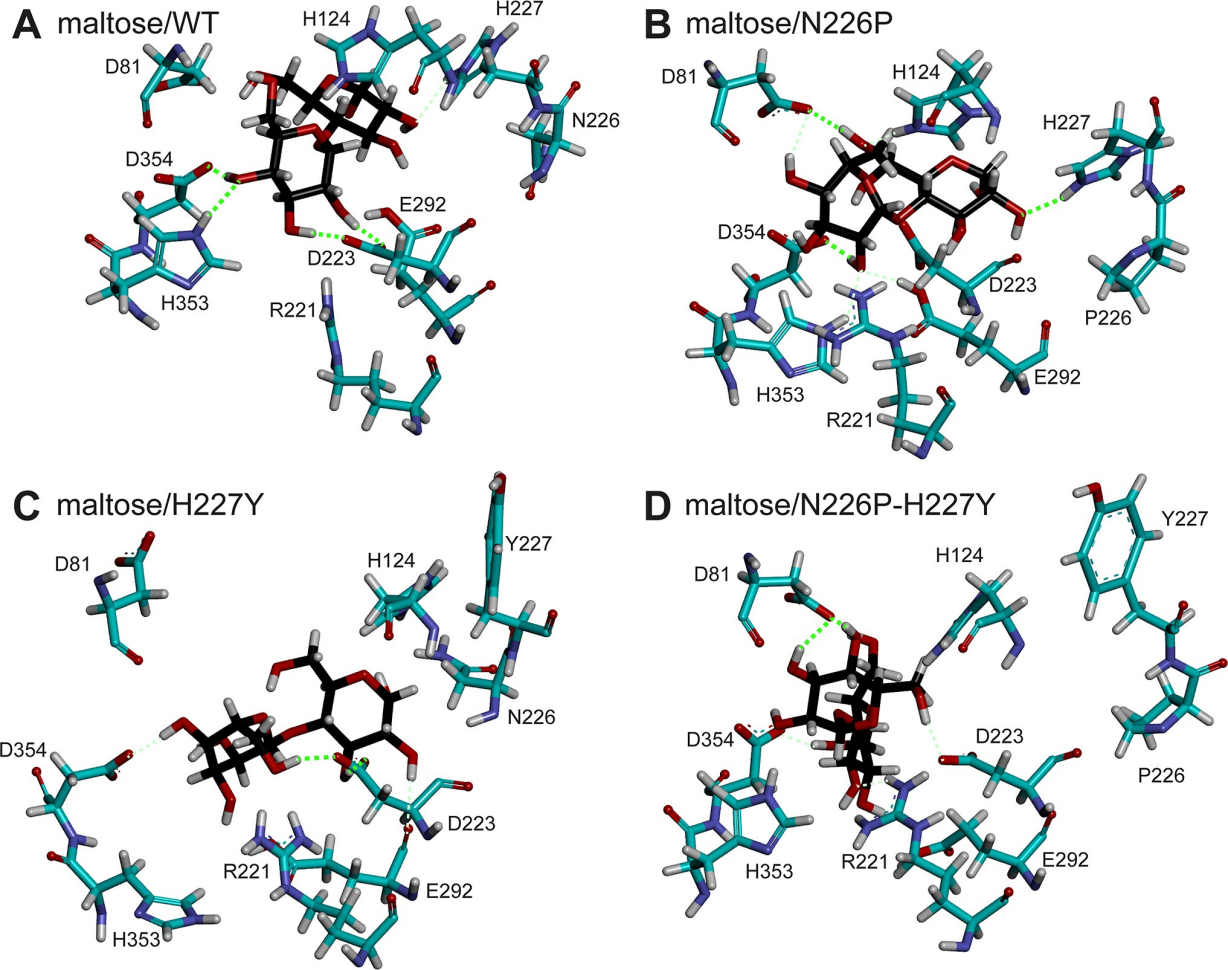
Examples of hydrogen bonds in A) maltose/WT, B) maltose/N226P, C) maltose/H227Y, and D) maltose/N226P-H227Y complexes during the 40–60 ns trajectories. Strong and medium hydrogen bonds are shown in neon green (thick dashed line) and light green (thin dashed line), respectively.

To identify the hydrogen bonds important for maltose binding, hydrogen bond occupations of all systems were calculated as shown in Table 3 and S9 Table. The number of strong and medium hydrogen bonds of the maltose/N226P complex is higher than that of the maltose/WT complex. Maltose has more favorable hydrogen bond interactions with WT than with H227Y and N226P-H227Y mutants. Although the maltose/H227Y complex has lower number of strong and medium hydrogen bonds than that of the maltose/N226P-H227Y complex, it has better overall electrostatic and van der Waals interactions than those of the maltose/N226P-H227Y complex (Table 1), causing maltose to bind better to the active site of the H227Y mutant than to that of the N226P-H227Y mutant. Examples of hydrogen bonds in all systems are shown in Fig 6.

**Table 3:** Number of strong and medium hydrogen bonds of the maltose/WT, maltose/N226P, maltose/H227Y, and maltose/N226P-H227Y complexes.

System	Number of strong and medium hydrogen bonds*	Binding residues that form hydrogen bonds with maltose
**Maltose/WT**	5 (4S, 1M)	D354 (S), D223 (2S), H353 (S), H227 (M)
**Maltose/N226P**	7 (3S, 4M)	D81 (S, M), D354 (S), H227 (S), H124 (M), E292 (M), H353 (M)
**Maltose/H227Y**	4 (2S, 2M)	D223 (2S), E292 (M), D354 (M)
**Maltose/N226P-H227Y**	5 (2S, 3M)	D81 (2S), D223 (M), R221 (M), D354 (M)

^*^S = Strong hydrogen bond, M = Medium hydrogen bond

### Substrate preference residue (residue 227)

Previous studies proposed residue 227 to be the substrate preference residue of HBGase III. Specifically, the wild-type HBGase III with Y227 preferred sucrose as a substrate, while the Y227H mutant preferred maltose as a substrate. Therefore, Y227 was proposed to be a sucrose preference residue, while H227 was proposed to be a maltose preference residue [2,3]. This residue is equivalent to H227 of HBGase II. Table 2 shows that H227 is an important maltose binding residue for both WT and the N226P mutant. However, when H227 was mutated to Y227 in H227Y and N226P-H227Y mutants, residue 227 is no longer an important maltose binding residue. Moreover, Table 3 shows that H227 formed medium and strong hydrogen bonds with maltose for WT and the N226P mutant, respectively, while Y227 did not form a hydrogen bond with maltose for both H227Y and N226P-H227Y mutants. These findings support the role of H227 as a maltose preference residue as proposed by previous studies.

**Fig 7:**
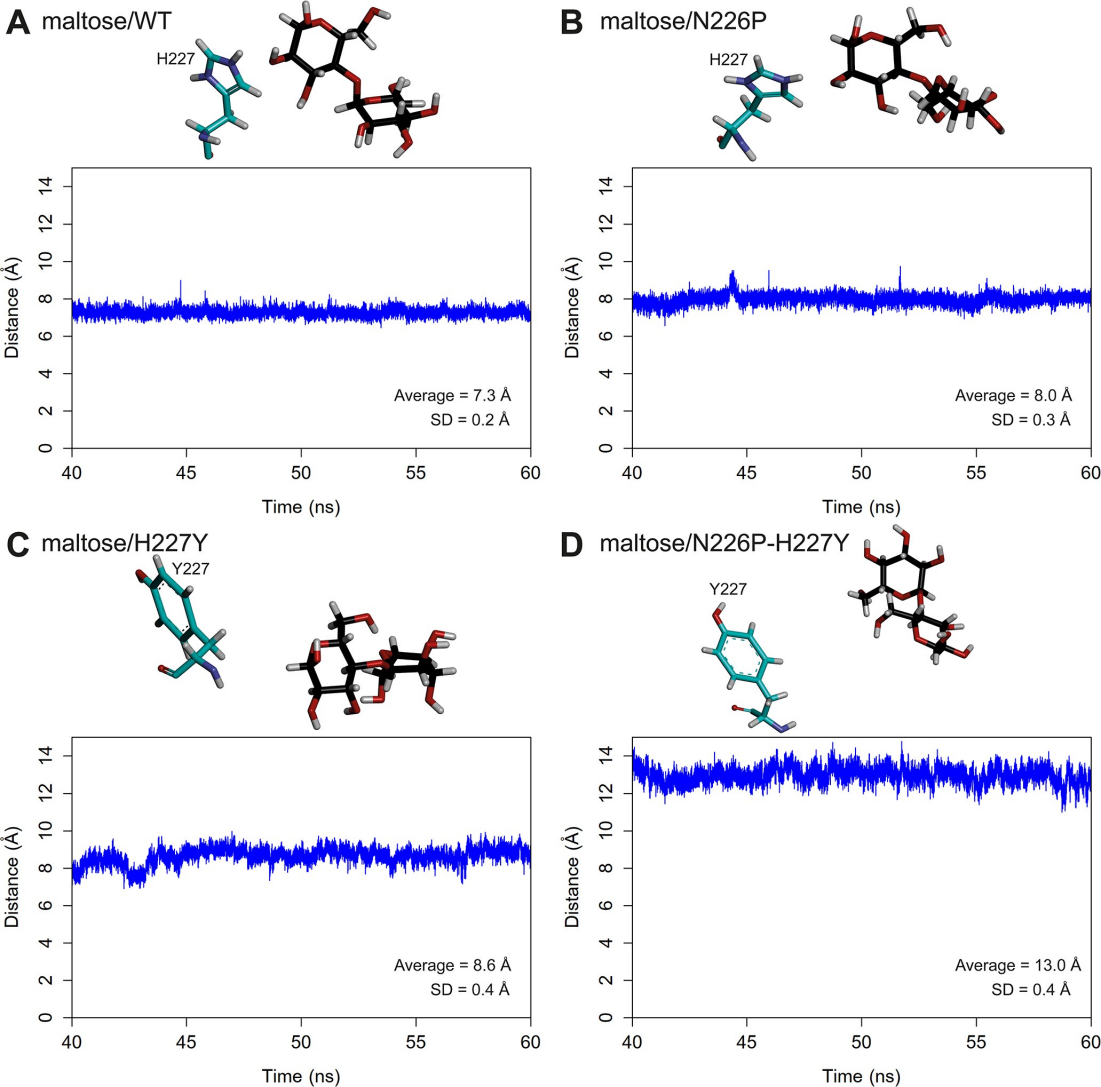
Distances between the centers of masses of the glucosyl residue at the reducing end of maltose and H227/Y227 of A) maltose/WT B) maltose/N226P C) maltose/H227Y and D) maltose/N226P-H227Y complexes.

To further elucidate the importance of this residue in maltose binding in the wild-type and mutant complexes, the distances between the centers of masses of the glucosyl residue of the reducing end of maltose and H227/Y227 were measured as shown in Fig 7. The mean distances between the glucosyl residue and H227 (7.3 Å for maltose/WT complex and 8.0 Å for maltose/N226P complex) were less than those between the glucosyl residue and Y227 (8.6 Å for maltose/H227Y complex and 13.0 Å for maltose/N226P-H227Y complex). These results suggest that H227 probably bound better to maltose than Y227, supporting the role of H227 as a maltose preference residue.

In terms of the binding free energy of residue 227, $\Delta G_{\mathrm{bind}}^{\mathrm{residue}}$ of H227 in maltose/WT and maltose/N226P complexes are more favorable than that of Y227 in maltose/H227Y and maltose/N226P-H227Y complexes (Table 4). In other words, the results suggest that H227 binds to maltose better than Y227 does, supporting the role of H227 as a substrate preference residue, specifically as a maltose preference residue [3]. The electrostatic interaction provides the main contribution to the favorable binding between maltose and H227. This result is also supported by the presence of a hydrogen bond between the glucosyl residue of maltose and H227 (67.1% occupancy in maltose/WT complex and 75.2% occupancy in maltose/N226P complex).

**Table 4:** Binding free energy decomposition of residue 227.

System	Energy contribution (kcal/mol)					
	Internal	Van der Waals	Electrostatic	Polar solvation	Non-polar solvation	Total
**Maltose/WT**	0.00	-0.50	-7.85	5.22	-0.10	-3.23
**Maltose/N226P**	0.00	0.07	-7.16	4.33	-0.08	-2.84
**Maltose/H227Y**	0.00	-0.31	-0.04	0.13	-0.03	-0.25
**Maltose/N226P-H227Y**	0.00	-0.04	-0.06	0.09	0.00	-0.01

## Conclusions

In this study, MD of the catalytically competent binding conformations of maltose/WT, maltose/N226P, maltose/H227Y, and maltose/N226P-H227Y complexes were performed at the experimental temperature and pH to elucidate the effects of N226P and H227Y mutations on the binding of maltose in the active site of *Apis mellifera* HBGase II. The results from our study support the hypothesis that the N226P mutation probably cause maltose to bind with better affinity and position/orientation for hydrolysis than WT, while the H227Y and N226P-H227Y mutations probably cause maltose to bind with worse affinity and position/orientation for hydrolysis than WT. Our findings show that the rankings of binding free energies and the distances between atoms necessary for hydrolysis of all systems reasonably support the experimental ranking of catalytic efficiencies of maltose hydrolysis. Moreover, our results revealed that maltose bound with better affinity in the active site of the N226P mutant and was in more appropriate position/orientation for hydrolysis than that of WT because it had higher number of important binding residues, strong and medium hydrogen bonds as well as shorter distance between atoms necessary for hydrolysis than WT. However, the H227Y and N226P-H227Y mutants had lower number of important binding residues and strong hydrogen bonds as well as longer distance between atoms necessary for hydrolysis than WT, resulting in less maltose binding affinity and less preferable position/orientation for maltose hydrolysis than WT. Thus, they could not effectively catalyze the reaction at the same catalytic efficiencies as WT. Our results also support the role of H227 as a maltose preference residue that was proposed in previous studies. We found that maltose bound H227 better than Y227 because H227 had better hydrogen bond and electrostatic interactions with maltose than Y227. Our study reveals important and novel insight into the effects of N226P and H227Y mutations on the binding of maltose in the active site of HBGase II.

## Supporting information

S1 Fig Ramachandran plot of the homology model of *Apis mellifera* α-glucosidase II. (TIF)

S2 Fig Superimposition of free WT and maltose/WT complex. Residues 223, 292, 226, and 227 as well as maltose are shown in stick representation and colored by atom types, where carbon atoms of maltose are black. Ribbon and carbon atoms of amino acid are colored in green for free enzyme and in cyan for the complex.
(TIF)

S3 Fig Superimposition of free N226P and maltose/N226P complex. Residues 223, 292, 226, and 227 as well as maltose are shown in stick representation and colored by atom types, where carbon atoms of maltose are black. Ribbon and carbon atoms of amino acid are colored in green for free enzyme and in cyan for the complex.
(TIF)

S4 Fig Superimposition of free H227Y and maltose/H227Y complex. Residues 223, 292, 226, and 227 as well as maltose are shown in stick representation and colored by atom types, where carbon atoms of maltose are black. Ribbon and carbon atoms of amino acid are colored in green for free enzyme and in cyan for the complex.
(TIF)

S5 Fig Superimposition of free N226P-H227Y and maltose/N226P-H227Y complex. Residues 223, 292, 226, and 227 as well as maltose are shown in stick representation and colored by atom types, where carbon atoms of maltose are black. Ribbon and carbon atoms of amino acid are colored in green for free enzyme and in cyan for the complex.
(TIF)

S6 Fig Superimposition of free maltose and maltose in all complexes. Maltose are shown in stick representation and colored by atom types, where carbon atoms of free maltose and maltose in WT, N226P, H227Y, and N226P-H227Y complexes are colored in purple, pink, orange, green, and yellow, respectively.
(TIF)

S1 Table Clustering of maltose binding conformations of the maltose/WT system. (DOCX)

S2 Table Clustering of maltose binding conformations of the maltose/N226P system. (DOCX)

S3 Table Clustering of maltose binding conformations of the maltose/H227Y system. (DOCX)

S4 Table Clustering of maltose binding conformations of the maltose/N226P-H227Y system. (DOCX)

S5 Table Energy contributions of the binding residues during 40–60 ns of the simulations of the maltose/WT complex. (DOCX)

S6 Table Energy contributions of the binding residues during 40–60 ns of the simulations of the maltose/N226P complex. (DOCX)

S7 Table Energy contributions of the binding residues during 40–60 ns of the simulations of the maltose/H227Y complex. (DOCX)

S8 Table Energy contributions of the binding residues during 40–60 ns of the simulations of the maltose/N226P-H227Y complex. (DOCX)

S9 Table Hydrogen bond occupations of all maltose-HBGase II systems. (DOCX)

## References

[1] Kubota M, Tsuji M, Nishimoto M, Wongchawalit J, Okuyama M, Mori H, et al Localization of α-Glucosidases I, II, and III in Organs of European Honeybees, *Apis mellifera* L., and the Origin of α-Glucosidase in Honey. Biosci Biotechnol Biochem. 2004;68: 2346–2352. 10.1271/bbb.68.2346 15564675

[2] Ngiwsara L, Iwai G, Tagami T, Sato N, Nakai H, Okuyama M, et al Amino Acids in Conserved Region II Are Crucial to Substrate Specificity, Reaction Velocity, and Regioselectivity in the Transglucosylation of Honeybee GH-13 α-Glucosidases. Biosci Biotechnol Biochem. 2012;76: 1967–1974. 10.1271/bbb.120473 23047117

[3] Pramoj Na Ayutthaya P, Chanchao C, Chunsrivirot S. Insight into the substrate specificity change caused by the Y227H mutation of α-glucosidase III from the European honeybee (*Apis mellifera*) through molecular dynamics simulations. PLoS One. 2018;13: 1–17. 10.1371/journal.pone.0198484 29864156 PMC5986129

[4] Nishimoto M, Mori H, Moteki T, Takamura Y, Iwai G, Miyaguchi Y, et al Molecular Cloning of cDNAs and Genes for Three α-Glucosidases from European Honeybees, *Apis mellifera* L., and Heterologous Production of Recombinant Enzymes in *Pichia pastoris*. Biosci Biotechnol Biochem. 2007;71: 1703–1716. 10.1271/bbb.70125 17617712

[5] Madsen LR, Stanley S, Swann P, Oswald J. A Survey of Commercially Available Isomaltooligosaccharide-Based Food Ingredients. J Food Sci. 2017;82: 401–408. 10.1111/1750-3841.13623 28140467

[6] Huang Z, Li Z, Su Y, Zhu Y, Zeng W, Chen G, et al Continuous Production of Isomalto-oligosaccharides by Thermo-inactivated Cells of *Aspergillus niger* J2 with Coarse Perlite as an Immobilizing Material. Appl Biochem Biotechnol. Applied Biochemistry and Biotechnology; 2018;185: 1088–1099. 10.1007/s12010-018-2706-6 29435830

[7] Zhang F, Wang W, Bah FBM, Song C, Zhou Y, Ji L, et al Heterologous expression of a thermostable α-glucosidase from *Geobacillus* sp. Strain HTA-462 by Escherichia coli and its potential application for isomaltose–oligosaccharide synthesis. Molecules. 2019;24 10.3390/molecules24071413 30974879 PMC6479687

[8] Andriotis VME, Saalbach G, Waugh R, Field RA, Smith AM. The maltase involved in starch metabolism in barley endosperm is encoded by a single gene. PLoS One. 2016;11: 1–13. 10.1371/journal.pone.0151642 27011041 PMC4807107

[9] Bathgate GN. A review of malting and malt processing for whisky distillation. J Inst Brew. 2016;122: 197–211. 10.1002/jib.332

[10] Chiba S. Molecular Mechanism in α-Glucosidase and Glucoamylase. Biosci Biotechnol Biochem. Taylor & Francis; 1997;61: 1233–1239. 10.1271/bbb.61.1233 9301101

[11] Waterhouse A, Bertoni M, Bienert S, Studer G, Tauriello G, Gumienny R, et al SWISS-MODEL: Homology modelling of protein structures and complexes. Nucleic Acids Res. Oxford University Press; 2018;46: W296–W303. 10.1093/nar/gky427 29788355 PMC6030848

[12] Guex N, Peitsch MC, Schwede T. Automated comparative protein structure modeling with SWISS-MODEL and Swiss-PdbViewer: A historical perspective. Electrophoresis. 2009;30: S162–S173. 10.1002/elps.200900140 19517507

[13] Benkert P, Biasini M, Schwede T. Toward the estimation of the absolute quality of individual protein structure models. Bioinformatics. 2011;27: 343–350. 10.1093/bioinformatics/btq662 21134891 PMC3031035

[14] Xu Z, Li S, Li J, Li Y, Feng X, Wang R, et al The Structural Basis of *Erwinia rhapontici* Isomaltulose Synthase. PLoS One. 2013;8: 1–11. 10.1371/journal.pone.0074788 24069347 PMC3777934

[15] Lovell SC, Davis IW, Arendall WB, De Bakker PIW, Word JM, Prisant MG, et al Structure validation by Cα geometry: φ,ψ and Cβ deviation. Proteins Struct Funct Genet. 2003;50: 437–450. 10.1002/prot.10286 12557186

[16] Gordon JC, Myers JB, Folta T, Shoja V, Heath LS, Onufriev A. H++: A server for estimating pKas and adding missing hydrogens to macromolecules. Nucleic Acids Res. 2005;33: W368–W371. 10.1093/nar/gki464 15980491 PMC1160225

[17] Myers J, Grothaus G, Narayanan S, Onufriev A. A simple clustering algorithm can be accurate enough for use in calculations of pKs in macromolecules. Proteins Struct Funct Bioinforma. John Wiley & Sons, Ltd; 2006;63: 928–938. 10.1002/prot.20922 16493626

[18] Anandakrishnan R, Aguilar B, Onufriev A V. H++ 3.0: Automating pK prediction and the preparation of biomolecular structures for atomistic molecular modeling and simulations. Nucleic Acids Res. 2012;40: W537–W541. 10.1093/nar/gks375 22570416 PMC3394296

[19] Case DA, Ben-Shalom IY, Brozell SR, Cerutti DS, Cheatham TEI, Cruzeiro VWD, et al AMBER 2018. San Francisco: University of California; 2018.

[20] Sitthiyotha T, Pichyangkura R, Chunsrivirot S. Molecular dynamics provides insight into how N251A and N251Y mutations in the active site of Bacillus licheniformis RN-01 levansucrase disrupt production of long-chain levan. PLoS One. 2018;13: 1–18. 10.1371/journal.pone.0204915 30278092 PMC6168164

[21] Shen X, Saburi W, Gai Z, Kato K, Ojima-Kato T, Yu J, et al Structural analysis of the α-glucosidase HaG provides new insights into substrate specificity and catalytic mechanism. Acta Crystallogr Sect D. 2015;71: 1382–1391. 10.1107/S139900471500721X 26057678

[22] Kirschner KN, Yongye AB, Tschampel SM, González-Outeiriño J, Daniels CR, Foley BL, et al GLYCAM06: A generalizable biomolecular force field. Carbohydrates. J Comput Chem. John Wiley & Sons, Ltd; 2008;29: 622–655. 10.1002/jcc.20820 17849372 PMC4423547

[23] Trott O, Olson AJ. AutoDock Vina: Improving the speed and accuracy of docking with a new scoring function, efficient optimization, and multithreading. J Comput Chem. John Wiley & Sons, Ltd; 2010;31: 455–461. 10.1002/jcc.21334 19499576 PMC3041641

[24] Feig M, Karanicolas J, Brooks CL. MMTSB Tool Set: enhanced sampling and multiscale modeling methods for applications in structural biology. J Mol Graph Model. 2004;22: 377–395. 10.1016/j.jmgm.2003.12.005 15099834

[25] Götz AW, Williamson MJ, Xu D, Poole D, Le Grand S, Walker RC. Routine microsecond molecular dynamics simulations with AMBER on GPUs. 1. generalized born. J Chem Theory Comput. 2012;8: 1542–1555. 10.1021/ct200909j 22582031 PMC3348677

[26] Le Grand S, Götz AW, Walker RC. SPFP: Speed without compromise—A mixed precision model for GPU accelerated molecular dynamics simulations. Comput Phys Commun. Elsevier B.V.; 2013;184: 374–380. 10.1016/j.cpc.2012.09.022

[27] Salomon-Ferrer R, Götz AW, Poole D, Le Grand S, Walker RC. Routine microsecond molecular dynamics simulations with AMBER on GPUs. 2. Explicit solvent particle mesh ewald. J Chem Theory Comput. 2013;9: 3878–3888. 10.1021/ct400314y 26592383

[28] Ryckaert JP, Ciccotti G, Berendsen HJC. Numerical integration of the cartesian equations of motion of a system with constraints: molecular dynamics of n-alkanes. J Comput Phys. 1977;23: 327–341. 10.1016/0021-9991(77)90098-5

[29] Swanson JMJ, Henchman RH, Mccammon JA. Revisiting Free Energy Calculations: A Theoretical Connection to MM/PBSA and Direct Calculation of the Association Free Energy. Biophys J. Elsevier; 2004;86: 67–74. 10.1016/S0006-3495(04)74084-9 14695250 PMC1303837

[30] Miller BR, McGee TD, Swails JM, Homeyer N, Gohlke H, Roitberg AE. MMPBSA.py: An efficient program for end-state free energy calculations. J Chem Theory Comput. 2012;8: 3314–3321. 10.1021/ct300418h 26605738

[31] R Core Team. R: A Language and Environment for Statistical Computing. Vienna: R Foundation for Statistical Computing; 2019.

[32] Letunic I, Bork P. Interactive Tree Of Life (iTOL) v4: recent updates and new developments. Nucleic Acids Res. 2019;47: W256–W259. 10.1093/nar/gkz239 30931475 PMC6602468

[33] Roe DR, Cheatham TE. PTRAJ and CPPTRAJ: Software for Processing and Analysis of Molecular Dynamics Trajectory Data. J Chem Theory Comput. 2013;9: 3084–95. 10.1021/ct400341p 26583988

