# Supplementary figures and images for: Molecular dynamics reveals insight into how N226P and H227Y mutations affect maltose binding in the active site of α-glucosidase II from European honeybee, Apis mellifera
Source: PLoS One. 2020 Mar 3;15(3):e0229734. doi: 10.1371/journal.pone.0229734 (PMC7053764; doi:10.1371/journal.pone.0229734)

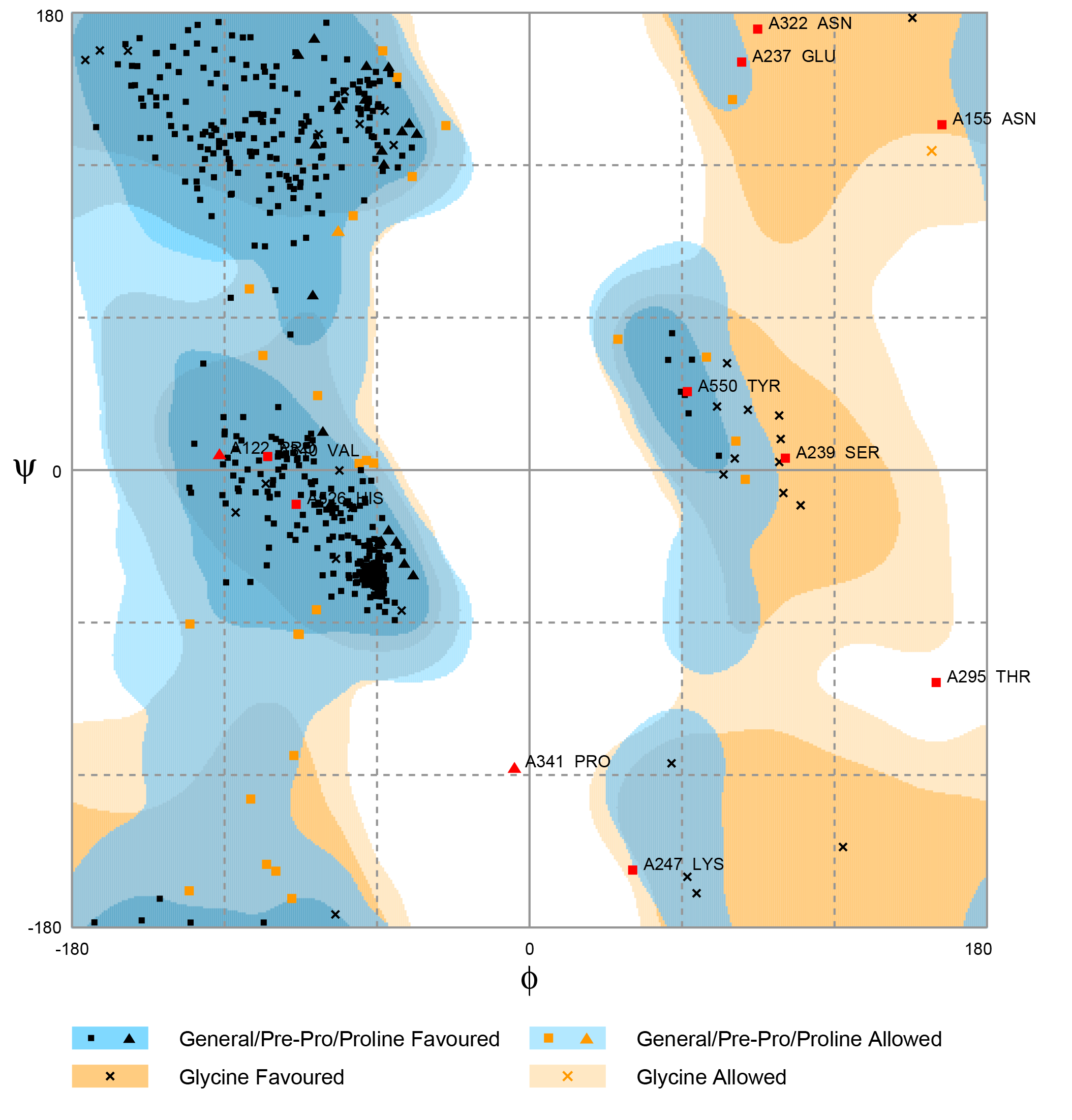

Supplement: S1 Fig — (TIF) [file pone.0229734.s001.tif]

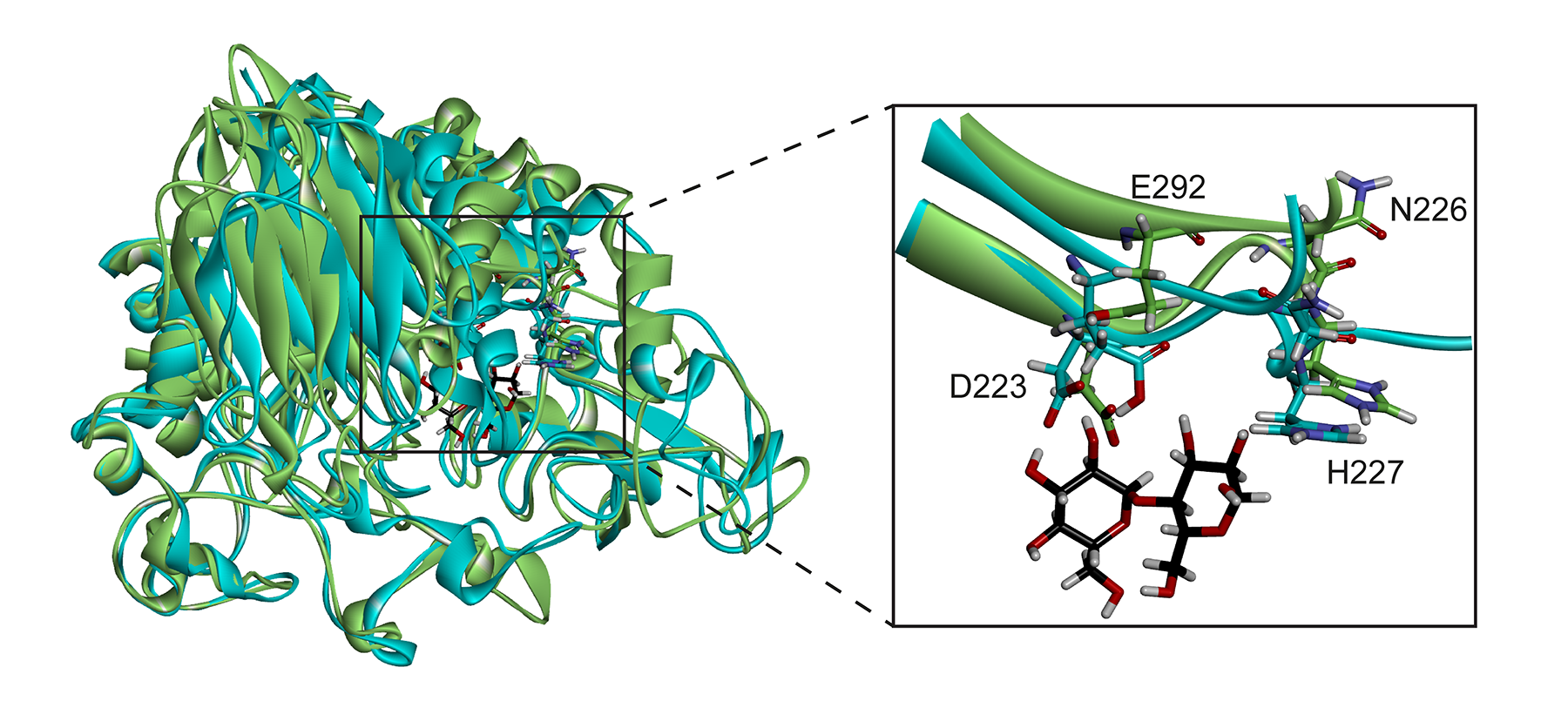

Supplement: S2 Fig — Residues 223, 292, 226, and 227 as well as maltose are shown in stick representation and colored by atom types, where carbon atoms of maltose are black. Ribbon and carbon atoms of amino acid are colored in green for free enzyme and in cyan for the complex. (TIF) [file pone.0229734.s002.tif]

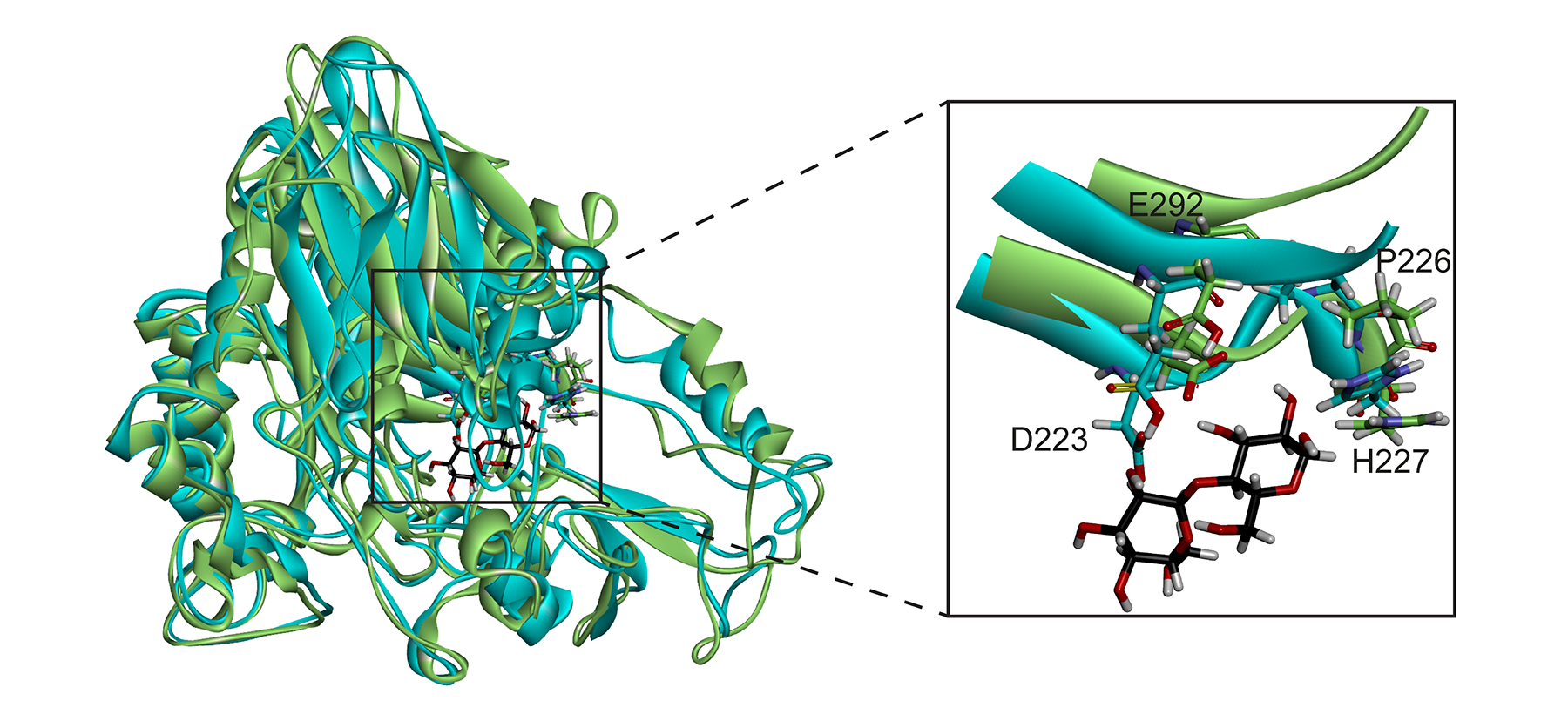

Supplement: S3 Fig — Residues 223, 292, 226, and 227 as well as maltose are shown in stick representation and colored by atom types, where carbon atoms of maltose are black. Ribbon and carbon atoms of amino acid are colored in green for free enzyme and in cyan for the complex. (TIF) [file pone.0229734.s003.tif]

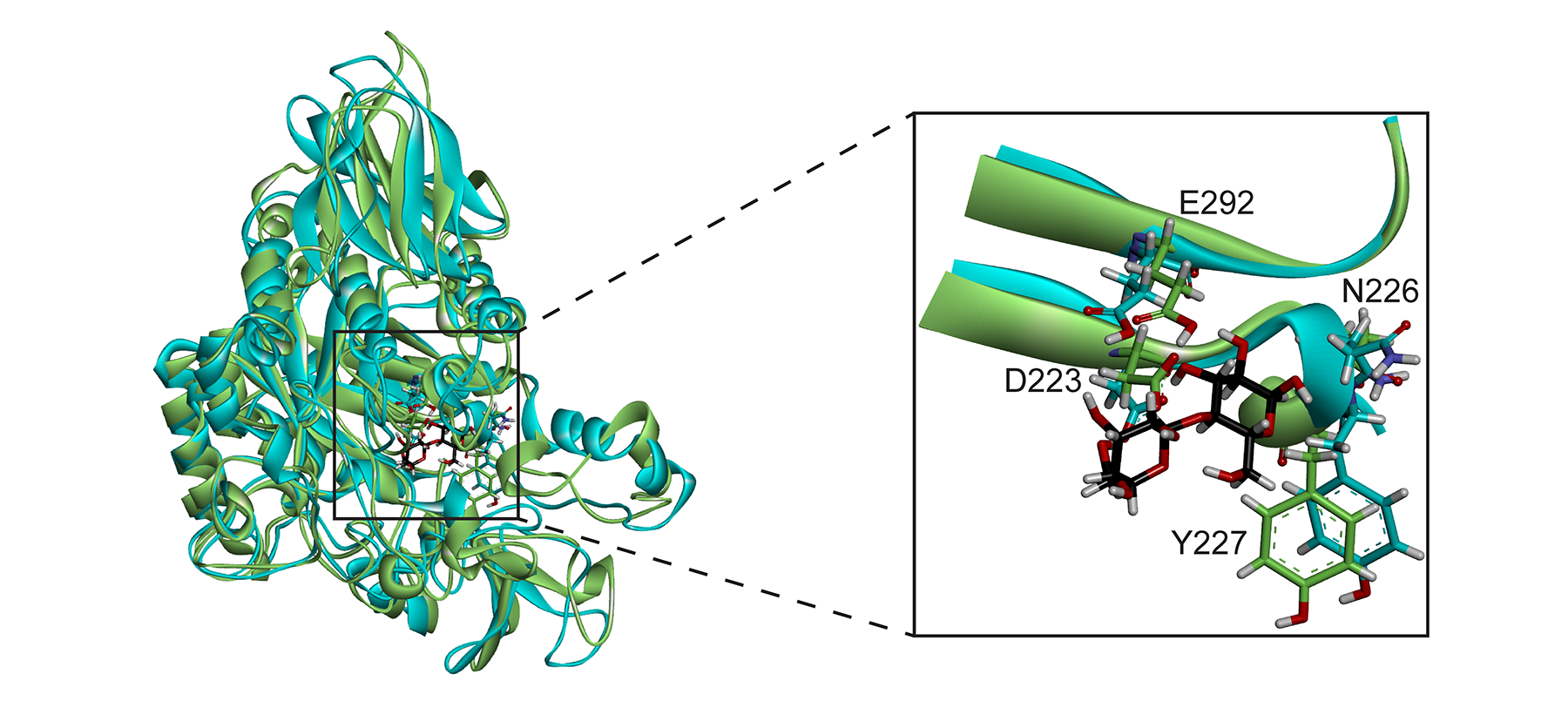

Supplement: S4 Fig — Residues 223, 292, 226, and 227 as well as maltose are shown in stick representation and colored by atom types, where carbon atoms of maltose are black. Ribbon and carbon atoms of amino acid are colored in green for free enzyme and in cyan for the complex. (TIF) [file pone.0229734.s004.tif]

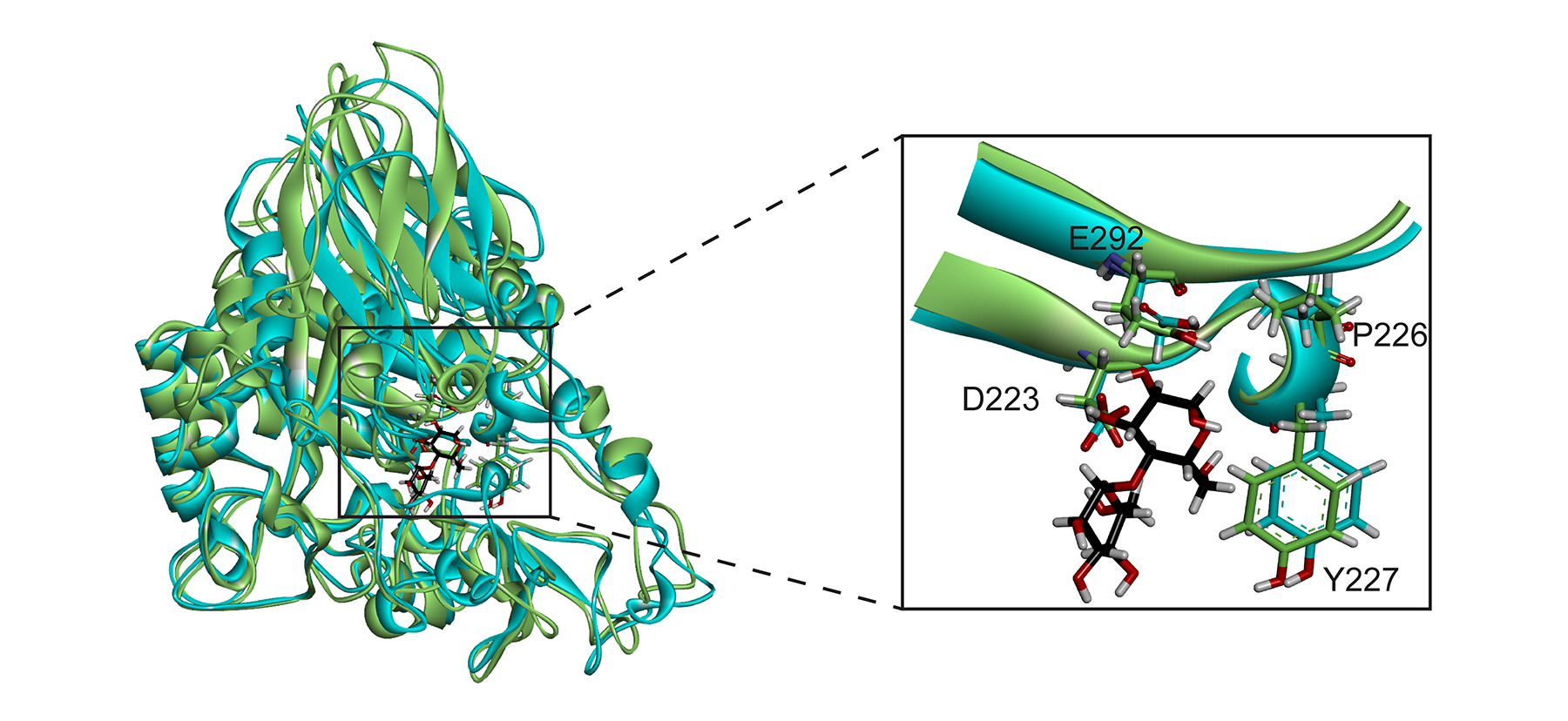

Supplement: S5 Fig — Residues 223, 292, 226, and 227 as well as maltose are shown in stick representation and colored by atom types, where carbon atoms of maltose are black. Ribbon and carbon atoms of amino acid are colored in green for free enzyme and in cyan for the complex. (TIF) [file pone.0229734.s005.tif]

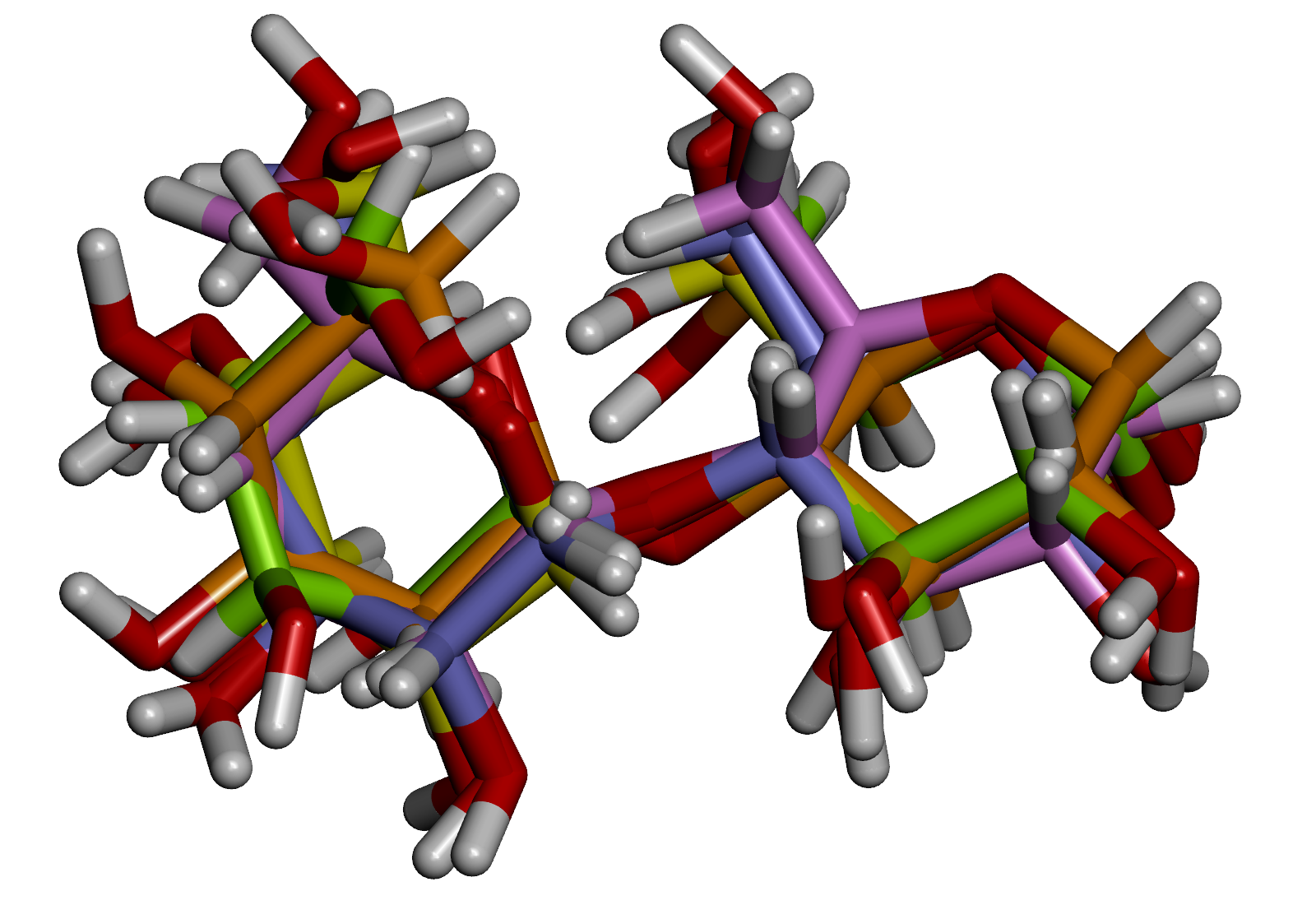

Supplement: S6 Fig — Maltose are shown in stick representation and colored by atom types, where carbon atoms of free maltose and maltose in WT, N226P, H227Y, and N226P-H227Y complexes are colored in purple, pink, orange, green, and yellow, respectively. (TIF) [file pone.0229734.s006.tif]
