# Supplementary material for: Molecular dynamics reveals insight into how N226P and H227Y mutations affect maltose binding in the active site of α-glucosidase II from European honeybee, Apis mellifera
Source: PLoS One. 2020 Mar 3;15(3):e0229734. doi: 10.1371/journal.pone.0229734 (PMC7053764; doi:10.1371/journal.pone.0229734)
Supplement: S1 Table — (DOCX) [file pone.0229734.s007.docx]

**S1 Table. Clustering of maltose binding conformations of the maltose/WT system.**

| **Cluster** | **No. of Members** | **Representative conformation*** | **Affinity**  **(kcal/mol)** | **Selected as catalytically competent binding conformation** |
| --- | --- | --- | --- | --- |
| 1 | 14 | 07-1 | -7.8 | Yes |
|  |  | 15-1 | -7.8 | No^♯^ |
|  |  | 20-1 | -7.8 | No^♯^ |
| 2 | 18 | 16-2 | -7.2 | No^♯^ |
|  |  | 15-2 | -7.1 | No^♯^ |
|  |  | 09-1 | -6.9 | No^♯^ |
| 3 | 13 | 08-6 | -6.8 | No^♯^ |
|  |  | 16-6 | -6.8 | No^♯^ |
|  |  | 02-5 | -6.5 | No^♯^ |
| 4 | 2 | 10-2 | -7.3 | No^♯^ |
| 5 | 6 | 12-1 | -7.9 | No^♯^ |
| 6 | 1 | 05-9 | -6.3 | No^♯^ |
| 7 | 1 | 17-8 | -6.7 | No^♯^ |
| 8 | 2 | 13-9 | -6.4 | No^♯^ |
| 9 | 1 | 17-7 | -6.7 | No^♯^ |

*Representative conformations are presented in the format m-n, representing the n^th^ binding conformation from the m^th^ docking run.

^♯^High O4-HE distance after MD
