# Supplementary material for: Molecular dynamics reveals insight into how N226P and H227Y mutations affect maltose binding in the active site of α-glucosidase II from European honeybee, Apis mellifera
Source: PLoS One. 2020 Mar 3;15(3):e0229734. doi: 10.1371/journal.pone.0229734 (PMC7053764; doi:10.1371/journal.pone.0229734)
Supplement: S3 Table — (DOCX) [file pone.0229734.s009.docx]

**S3 Table. Clustering of maltose binding conformations of the maltose/H227Y system.**

| **Cluster** | **No. of Members** | **Representative conformation*** | **Affinity**  **(kcal/mol)** | **Selected as catalytically competent binding conformation** |
| --- | --- | --- | --- | --- |
| 1 | 27 | 16-1 | -7.7 | No^♯^ |
|  |  | 01-1 | -7.7 | No^♯^ |
|  |  | 06-1 | -7.7 | No^♯^ |
| 2 | 19 | 03-2 | -7.2 | No^♯^ |
|  |  | 08-2 | -7.1 | No^♯^ |
|  |  | 16-2 | -7.1 | No^♯^ |
| 3 | 15 | 14-4 | -6.6 | No^♯^ |
|  |  | 04-7 | -6.5 | No^♯^ |
|  |  | 11-5 | -6.8 | No^♯^ |
| 4 | 7 | 16-7 | -6.5 | Yes |
| 5 | 2 | 15-3 | -6.9 | No^♯^ |
| 6 | 1 | 10-8 | -6.7 | No^†^ |
| 7 | 1 | 20-5 | -7.0 | No^♯^ |
| 8 | 1 | 15-8 | -6.6 | No^♯^ |
| 9 | 1 | 17-6 | -6.6 | No^♯^ |
| 10 | 1 | 18-9 | -6.2 | No^†^ |

*Representative conformations are presented in the format m-n, representing the n^th^ binding conformation from the m^th^ docking run.

^♯^High O4-HE distance after MD

^†^RMSD values are not stable and not reasonable.
