# Supplementary material for: Molecular dynamics reveals insight into how N226P and H227Y mutations affect maltose binding in the active site of α-glucosidase II from European honeybee, Apis mellifera
Source: PLoS One. 2020 Mar 3;15(3):e0229734. doi: 10.1371/journal.pone.0229734 (PMC7053764; doi:10.1371/journal.pone.0229734)
Supplement: S4 Table — (DOCX) [file pone.0229734.s010.docx]

**S4 Table. Clustering of maltose binding conformations of the maltose/N226P-H227Y system.**

| **Cluster** | **No. of Members** | **Representative conformation*** | **Affinity**  **(kcal/mol)** | **Selected as catalytically competent binding conformation** |
| --- | --- | --- | --- | --- |
| 1 | 22 | 07-1 | -7.7 | No^♯^ |
|  |  | 03-1 | -7.7 | No^‡^ |
|  |  | 11-1 | -7.7 | No^♯^ |
| 2 | 19 | 10-2 | -7.2 | No^‡^ |
|  |  | 17-2 | -7.1 | No^♯^ |
|  |  | 20-2 | -7.1 | Yes |
| 3 | 7 | 06-4 | -6.8 | No^♯^ |
| 4 | 1 | 03-6 | -6.7 | No^‡^ |
| 5 | 6 | 09-9 | -6.5 | No^♯^ |
| 6 | 9 | 15-9 | -6.5 | No^♯^ |
| 7 | 2 | 14-3 | -7.1 | No^♯^ |

*Representative conformations are presented in the format m-n, representing the n^th^ binding conformation from the m^th^ docking run.

^♯^High O4-HE distance after MD

^‡^High fluctuation of O4-HE distance after MD
