# Supplementary material for: Molecular dynamics reveals insight into how N226P and H227Y mutations affect maltose binding in the active site of α-glucosidase II from European honeybee, Apis mellifera
Source: PLoS One. 2020 Mar 3;15(3):e0229734. doi: 10.1371/journal.pone.0229734 (PMC7053764; doi:10.1371/journal.pone.0229734)
Supplement: S5 Table — (DOCX) [file pone.0229734.s011.docx]

**S5 Table. Energy contributions of the binding residues during 40-60 ns of the simulations of the maltose/WT complex.**

| **Residue** | **Energy contribution (kcal/mol)** | | | | | |
| --- | --- | --- | --- | --- | --- | --- |
|  | **Internal** | **Van der Waals** | **Electrostatic** | **Polar solvation** | **Non-polar solvation** | **Total** |
| 81 | 0.00 | -0.07 | -7.06 | 6.19 | -0.04 | -0.99 |
| 84 | 0.00 | -2.01 | 0.96 | 0.06 | -0.07 | -1.07 |
| 121 | 0.00 | -0.65 | 0.45 | -0.42 | -0.05 | -0.66 |
| 124 | 0.00 | -0.54 | -2.58 | 2.25 | -0.06 | -0.93 |
| 167 | 0.00 | -0.74 | -0.15 | 0.17 | -0.13 | -0.85 |
| 168 | 0.00 | -0.69 | -0.10 | 0.16 | -0.12 | -0.75 |
| 186 | 0.00 | -0.10 | -0.09 | 0.13 | 0.00 | -0.06 |
| 187 | 0.00 | -1.95 | -0.13 | 0.38 | -0.20 | -1.90 |
| 188 | 0.00 | -0.05 | 0.05 | -0.06 | 0.00 | -0.06 |
| 191 | 0.00 | -0.30 | 0.73 | -0.59 | 0.00 | -0.16 |
| 221 | 0.00 | -0.44 | 3.33 | -4.23 | 0.00 | -1.35 |
| 223 | 0.00 | 0.94 | -16.10 | 12.74 | -0.06 | -2.48 |
| 224 | 0.00 | -1.07 | 0.35 | -0.71 | -0.08 | -1.51 |
| 225 | 0.00 | -0.09 | 0.08 | -0.05 | 0.00 | -0.06 |
| 226 | 0.00 | -0.20 | -0.40 | 0.39 | 0.00 | -0.22 |
| 227 | 0.00 | -0.50 | -7.85 | 5.22 | -0.10 | -3.23 |
| 255 | 0.00 | -0.13 | 0.33 | -0.24 | 0.00 | -0.04 |
| 258 | 0.00 | 0.00 | -0.01 | 0.02 | 0.00 | 0.00 |
| 259 | 0.00 | 0.00 | -0.26 | 0.26 | 0.00 | 0.00 |
| 292 | 0.00 | -0.55 | -0.81 | 0.44 | -0.07 | -0.99 |
| 294 | 0.00 | -1.33 | -0.52 | 0.97 | -0.12 | -1.00 |
| 295 | 0.00 | -0.05 | 0.00 | 0.07 | 0.00 | 0.02 |
| 314 | 0.00 | -2.47 | 0.15 | 0.68 | -0.27 | -1.90 |
| 317 | 0.00 | -0.20 | -0.02 | -0.02 | 0.00 | -0.24 |
| 352 | 0.00 | -0.17 | -0.33 | 0.33 | 0.00 | -0.17 |
| 353 | 0.00 | -0.12 | -3.71 | 0.36 | -0.05 | -3.52 |
| 354 | 0.00 | -0.42 | -11.35 | 10.88 | -0.11 | -1.00 |
| 355 | 0.00 | -0.04 | -0.13 | 0.19 | 0.00 | 0.02 |
| 405 | 0.00 | -0.11 | 0.02 | -0.02 | -0.01 | -0.11 |
| 417 | 0.00 | -0.05 | -0.25 | 0.32 | -0.01 | 0.01 |
| 419 | 0.00 | -1.16 | 3.98 | -4.51 | -0.10 | -1.79 |
| 423 | 0.00 | -0.28 | 1.15 | -1.80 | 0.00 | -0.92 |
