# Supplementary material for: Molecular dynamics reveals insight into how N226P and H227Y mutations affect maltose binding in the active site of α-glucosidase II from European honeybee, Apis mellifera
Source: PLoS One. 2020 Mar 3;15(3):e0229734. doi: 10.1371/journal.pone.0229734 (PMC7053764; doi:10.1371/journal.pone.0229734)
Supplement: S6 Table — (DOCX) [file pone.0229734.s012.docx]

**S6 Table. Energy contributions of the binding residues during 40-60 ns of the simulations of the maltose/N226P complex.**

| **Residue** | **Energy contribution (kcal/mol)** | | | | | |
| --- | --- | --- | --- | --- | --- | --- |
|  | **Internal** | **Van der Waals** | **Electrostatic** | **Polar solvation** | **Non-polar solvation** | **Total** |
| 81 | 0.00 | 0.73 | -20.47 | 18.11 | -0.04 | -1.67 |
| 84 | 0.00 | -2.21 | 0.17 | 0.64 | -0.04 | -1.44 |
| 121 | 0.00 | -0.22 | 0.02 | 0.00 | 0.00 | -0.20 |
| 124 | 0.00 | 0.16 | -2.33 | -0.66 | -0.02 | -2.85 |
| 167 | 0.00 | -0.86 | 0.10 | -0.07 | -0.17 | -1.00 |
| 168 | 0.00 | -0.90 | 0.19 | -0.09 | -0.08 | -0.87 |
| 186 | 0.00 | -0.18 | 0.05 | 0.03 | 0.00 | -0.11 |
| 187 | 0.00 | -2.31 | -0.26 | 0.36 | -0.18 | -2.39 |
| 188 | 0.00 | -0.05 | 0.18 | -0.19 | 0.00 | -0.06 |
| 191 | 0.00 | -0.44 | 0.91 | -0.52 | 0.00 | -0.05 |
| 221 | 0.00 | -0.04 | -6.64 | 1.90 | -0.03 | -4.80 |
| 223 | 0.00 | -1.31 | -7.36 | 8.82 | -0.10 | 0.06 |
| 224 | 0.00 | -0.88 | 0.15 | -0.73 | -0.04 | -1.51 |
| 225 | 0.00 | -0.09 | 0.05 | 0.00 | 0.00 | -0.04 |
| 226 | 0.00 | -0.18 | -0.08 | 0.07 | 0.00 | -0.19 |
| 227 | 0.00 | 0.07 | -7.16 | 4.33 | -0.08 | -2.84 |
| 255 | 0.00 | -0.01 | -0.31 | 0.32 | 0.00 | 0.01 |
| 258 | 0.00 | -0.01 | 0.08 | -0.07 | 0.00 | 0.00 |
| 259 | 0.00 | -0.01 | -0.48 | 0.49 | 0.00 | 0.00 |
| 292 | 0.00 | -1.16 | -3.29 | 2.91 | -0.10 | -1.64 |
| 294 | 0.00 | -0.98 | 0.12 | 0.06 | -0.15 | -0.96 |
| 295 | 0.00 | -0.05 | -0.01 | 0.06 | 0.00 | -0.01 |
| 314 | 0.00 | -2.55 | -0.52 | 0.93 | -0.23 | -2.38 |
| 317 | 0.00 | -0.15 | 0.02 | -0.06 | 0.00 | -0.19 |
| 352 | 0.00 | -0.10 | -0.01 | 0.10 | 0.00 | -0.01 |
| 353 | 0.00 | -0.67 | -2.89 | 0.67 | -0.01 | -2.90 |
| 354 | 0.00 | -0.24 | -7.93 | 9.60 | -0.08 | 1.35 |
| 355 | 0.00 | -0.04 | -0.07 | 0.13 | 0.00 | 0.03 |
| 405 | 0.00 | -0.05 | 0.00 | 0.00 | 0.00 | -0.04 |
| 417 | 0.00 | -0.03 | -0.05 | 0.11 | 0.00 | 0.04 |
| 419 | 0.00 | -1.52 | 1.16 | -3.90 | -0.12 | -4.37 |
| 423 | 0.00 | -0.57 | -0.98 | -0.79 | -0.01 | -2.35 |
