# Supplementary material for: Molecular dynamics reveals insight into how N226P and H227Y mutations affect maltose binding in the active site of α-glucosidase II from European honeybee, Apis mellifera
Source: PLoS One. 2020 Mar 3;15(3):e0229734. doi: 10.1371/journal.pone.0229734 (PMC7053764; doi:10.1371/journal.pone.0229734)
Supplement: S7 Table — (DOCX) [file pone.0229734.s013.docx]

**S7 Table. Energy contributions of the binding residues during 40-60 ns of the simulations of the maltose/H227Y complex.**

| **Residue** | **Energy contribution (kcal/mol)** | | | | | |
| --- | --- | --- | --- | --- | --- | --- |
|  | **Internal** | **Van der Waals** | **Electrostatic** | **Polar solvation** | **Non-polar solvation** | **Total** |
| 81 | 0.00 | -0.08 | -0.40 | 0.59 | 0.00 | 0.11 |
| 84 | 0.00 | -1.34 | -0.36 | 0.72 | -0.14 | -1.11 |
| 121 | 0.00 | -0.09 | 0.13 | -0.11 | 0.00 | -0.07 |
| 124 | 0.00 | -0.06 | 0.55 | -0.45 | 0.00 | 0.04 |
| 167 | 0.00 | -0.14 | 0.01 | 0.01 | -0.05 | -0.17 |
| 168 | 0.00 | -0.03 | 0.03 | -0.01 | 0.00 | -0.01 |
| 186 | 0.00 | -0.08 | 0.11 | -0.05 | 0.00 | -0.02 |
| 187 | 0.00 | -0.50 | -0.05 | 0.13 | -0.22 | -0.63 |
| 188 | 0.00 | -0.01 | -0.02 | 0.03 | 0.00 | 0.00 |
| 191 | 0.00 | -0.02 | 0.11 | -0.07 | 0.00 | 0.02 |
| 221 | 0.00 | -0.51 | -5.47 | -0.84 | -0.12 | -6.94 |
| 223 | 0.00 | 0.03 | -17.23 | 15.29 | -0.20 | -2.11 |
| 224 | 0.00 | -1.04 | 0.62 | -0.66 | -0.09 | -1.17 |
| 225 | 0.00 | -0.75 | -0.99 | -0.07 | -0.01 | -1.83 |
| 226 | 0.00 | -0.95 | -0.34 | 0.28 | -0.09 | -1.10 |
| 227 | 0.00 | -0.31 | -0.04 | 0.13 | -0.03 | -0.25 |
| 255 | 0.00 | -0.01 | -0.53 | 0.54 | 0.00 | 0.00 |
| 258 | 0.00 | -0.02 | 0.03 | -0.02 | 0.00 | -0.01 |
| 259 | 0.00 | -0.04 | -0.90 | 0.93 | 0.00 | -0.02 |
| 292 | 0.00 | -1.65 | -1.89 | 1.92 | -0.11 | -1.73 |
| 294 | 0.00 | -3.10 | -3.21 | 3.38 | -0.44 | -3.38 |
| 295 | 0.00 | -0.07 | 0.07 | 0.00 | 0.00 | 0.01 |
| 314 | 0.00 | -0.88 | -0.01 | 0.36 | -0.05 | -0.58 |
| 317 | 0.00 | -0.05 | -0.02 | 0.02 | 0.00 | -0.05 |
| 352 | 0.00 | -0.22 | -0.59 | 0.66 | -0.01 | -0.16 |
| 353 | 0.00 | -0.77 | -0.58 | 0.47 | -0.05 | -0.93 |
| 354 | 0.00 | 0.10 | -8.93 | 9.07 | -0.19 | 0.06 |
| 355 | 0.00 | -0.07 | -0.03 | 0.08 | 0.00 | -0.02 |
| 405 | 0.00 | -0.01 | 0.10 | -0.09 | 0.00 | 0.00 |
| 417 | 0.00 | -0.05 | -1.34 | 1.43 | 0.00 | 0.04 |
| 419 | 0.00 | -0.33 | -0.49 | 0.33 | -0.07 | -0.57 |
| 423 | 0.00 | -0.13 | 1.31 | -1.27 | -0.01 | -0.10 |
