# Supplementary material for: Molecular dynamics reveals insight into how N226P and H227Y mutations affect maltose binding in the active site of α-glucosidase II from European honeybee, Apis mellifera
Source: PLoS One. 2020 Mar 3;15(3):e0229734. doi: 10.1371/journal.pone.0229734 (PMC7053764; doi:10.1371/journal.pone.0229734)
Supplement: S8 Table — (DOCX) [file pone.0229734.s014.docx]

**S8 Table. Energy contributions of the binding residues during 40-60 ns of the simulations of the maltose/N226P-H227Y complex.**

| **Residue** | **Energy contribution (kcal/mol)** | | | | | |
| --- | --- | --- | --- | --- | --- | --- |
|  | **Internal** | **Van der Waals** | **Electrostatic** | **Polar solvation** | **Non-polar solvation** | **Total** |
| 81 | 0.00 | 0.89 | -14.84 | 13.34 | -0.15 | -0.78 |
| 84 | 0.00 | -2.84 | -0.01 | 0.32 | -0.14 | -2.66 |
| 121 | 0.00 | -0.33 | -0.03 | -0.03 | 0.00 | -0.39 |
| 124 | 0.00 | -0.41 | -5.42 | 3.21 | -0.03 | -2.65 |
| 167 | 0.00 | -0.25 | -0.01 | 0.04 | -0.07 | -0.29 |
| 168 | 0.00 | -0.35 | 0.02 | 0.05 | -0.06 | -0.34 |
| 186 | 0.00 | -0.06 | -0.13 | 0.15 | 0.00 | -0.05 |
| 187 | 0.00 | -1.51 | -0.05 | 0.12 | -0.25 | -1.69 |
| 188 | 0.00 | -0.07 | 0.14 | -0.15 | 0.00 | -0.08 |
| 191 | 0.00 | -0.47 | 0.78 | -0.35 | 0.00 | -0.04 |
| 221 | 0.00 | -0.89 | -3.39 | 0.20 | -0.17 | -4.26 |
| 223 | 0.00 | -0.27 | -3.32 | 3.91 | -0.07 | 0.25 |
| 224 | 0.00 | -0.24 | 0.00 | 0.01 | -0.01 | -0.23 |
| 225 | 0.00 | -0.03 | 0.01 | 0.00 | 0.00 | -0.01 |
| 226 | 0.00 | -0.02 | 0.01 | 0.00 | 0.00 | -0.02 |
| 227 | 0.00 | -0.04 | -0.06 | 0.09 | 0.00 | -0.01 |
| 255 | 0.00 | 0.00 | 0.11 | -0.09 | 0.00 | 0.01 |
| 258 | 0.00 | -0.01 | 0.01 | 0.00 | 0.00 | 0.00 |
| 259 | 0.00 | 0.00 | 0.03 | -0.02 | 0.00 | 0.00 |
| 292 | 0.00 | -0.98 | -0.04 | 0.71 | -0.16 | -0.47 |
| 294 | 0.00 | -0.28 | -0.03 | 0.19 | -0.06 | -0.18 |
| 295 | 0.00 | -0.01 | 0.00 | 0.01 | 0.00 | 0.00 |
| 314 | 0.00 | -0.36 | 0.02 | 0.05 | -0.09 | -0.37 |
| 317 | 0.00 | -0.42 | -0.01 | 0.04 | -0.15 | -0.55 |
| 352 | 0.00 | -0.31 | -0.75 | 1.20 | -0.06 | 0.08 |
| 353 | 0.00 | -0.75 | -2.45 | 1.20 | -0.08 | -2.08 |
| 354 | 0.00 | -0.89 | -8.74 | 9.66 | -0.33 | -0.31 |
| 355 | 0.00 | -0.15 | -0.18 | 0.26 | -0.02 | -0.09 |
| 405 | 0.00 | -0.01 | 0.01 | -0.01 | 0.00 | -0.01 |
| 417 | 0.00 | -0.03 | 0.19 | -0.13 | 0.00 | 0.03 |
| 419 | 0.00 | -0.43 | 0.69 | -0.57 | -0.08 | -0.39 |
| 423 | 0.00 | -0.27 | -0.47 | -0.02 | -0.04 | -0.81 |
