# Supplementary material for: Molecular dynamics reveals insight into how N226P and H227Y mutations affect maltose binding in the active site of α-glucosidase II from European honeybee, Apis mellifera
Source: PLoS One. 2020 Mar 3;15(3):e0229734. doi: 10.1371/journal.pone.0229734 (PMC7053764; doi:10.1371/journal.pone.0229734)
Supplement: S9 Table — (DOCX) [file pone.0229734.s015.docx]

**S9 Table. Hydrogen bond occupations of each maltose-HBGase II system**

| **System** | **Acceptor** | **DonorH** | **Occupancy**^*^ **(%)** |
| --- | --- | --- | --- |
| **Maltose/WT** | D354@OD2 | 0GA@H4O | 96.13 (S) |
|  | D223@OD2 | 0GA@H3O | 94.22 (S) |
|  | D223@OD1 | 0GA@H2O | 78.38 (S) |
|  | 0GA@O4 | H353@HE2 | 75.47 (S) |
|  | 4GA@O2 | H227@HE2 | 67.13 (M) |
| **Maltose/N226P** | D81@OD2 | 0GA@H6O | 99.79 (S) |
|  | D354@OD1 | 0GA@H2O | 97.72 (S) |
|  | 4GA@O2 | H227@HE2 | 75.17 (S) |
|  | D81@OD2 | 0GA@H4O | 67.24 (M) |
|  | 0GA@O6 | H124@HE2 | 62.36 (M) |
|  | 0GA@O2 | E292@HE2 | 59.71 (M) |
|  | 0GA@O2 | H353@HE2 | 54.23 (M) |
| **Maltose/H227Y** | D223@OD2 | 4GA@H3O | 97.38 (S) |
|  | D223@OD1 | 0GA@H6O | 85.15 (S) |
|  | E292@O | 4GA@H2O | 65.81 (M) |
|  | D354@OD1 | 0GA@H4O | 62.50 (M) |
| **Maltose/N226P-H227Y** | D81@OD2 | 0GA@H4O | 99.10 (S) |
|  | D81@OD2 | 0GA@H3O | 88.64 (S) |
|  | D223@OD1 | 0GA@H6O | 64.82 (M) |
|  | 4GA@O3 | R221@HH21 | 64.00 (M) |
|  | D354@OD1 | ROH@HO1 | 50.01 (M) |

^*^Only hydrogen bonds with the occupations of more than 50% are shown: S = strong hydrogen bond and M = medium hydrogen bond.
